# Understanding of complex spin up-conversion processes in charge-transfer-type organic molecules

**DOI:** 10.1038/s41467-024-46406-5

**Published:** 2024-03-13

**Authors:** Hyung Suk Kim, Sang Hoon Lee, Seunghyup Yoo, Chihaya Adachi

**Affiliations:** 1 Center for Organic Photonics and Electronics Research (OPERA), Kyushu University, 744 Motooka, Nishi, Fukuoka, 819-0395 Japan; 2 Department of Applied Chemistry, Kyushu University, 744 Motooka, Nishi, Fukuoka, 819-0395 Japan; 3 School of Electrical Engineering, Korea Advanced Institute of Science and Technology (KAIST), Daejeon, 34141 Republic of Korea; 4 International Institute for Carbon Neutral Energy Research (I2CNER), Kyushu University, 744 Motooka, Nishi, Fukuoka, 819-0395 Japan

**Keywords:** Chemical physics, Materials for devices

## Abstract

Despite significant progress made over the past decade in thermally activated delayed fluorescence (TADF) molecules as a material paradigm for enhancing the performance of organic light-emitting diodes, the underlying spin-flip mechanism in these charge-transfer (CT)-type molecular systems remains an enigma, even since its initial report in 2012. While the initial and final electronic states involved in spin-flip between the lowest singlet and lowest triplet excited states are well understood, the exact dynamic processes and the role of intermediate high-lying triplet (T) states are still not fully comprehended. In this context, we propose a comprehensive model to describe the spin-flip processes applicable for a typical CT-type molecule, revealing the origin of the high-lying T state in a partial molecular framework in CT-type molecules. This work provides experimental and theoretical insights into the understanding of intersystem crossing for CT-type molecules, facilitating more precise control over spin-flip rates and thus advancing toward developing the next-generation platform for purely organic luminescent candidates.

## Abstract (web summary)

Kim et al. propose a model for the complex spin-flip process in charge-transfer-type thermally activated delayed fluorescence molecules, uncovering the origin of the high-lying triplet state, addressing a pressing issue discussed over the last decade.

## Introduction

Advances in organic optoelectronics have been driven by a deeper understanding of the exciton dynamic processes that enable the conversion between excited states with different spin multiplicities^1–5^. One promising approach is to use organic luminescent materials that exhibit thermally activated delayed fluorescence (TADF), which involves a spin-flip mechanism comprising both forward and reverse intersystem crossings (i.e., ISC and RISC). This process is achieved by bringing the low-lying singlet (S_1_) and triplet (T_1_) manifolds close enough in energy to promote efficient interconversion for exo- and endothermic ISC between them, thereby generating photons as a form of delayed fluorescence^1^. To do so, one can promote a spatial charge separation, known as charge-transfer (CT), between the highest occupied molecular orbital (HOMO) and the lowest unoccupied molecular orbital (LUMO), leading to the minimal energy difference between the two lowest excited states (ΔE_ST_)^6–9^. With this approach, the triplet spin-uphill efficiency for CT-type TADF molecules reported to date has reached a value close to the unity^10–12^. However, the quantum-mechanical solution to the small exchange energy (*K*) required for low ΔE_ST_ mandates that most TADF molecular systems have both S_1_ and T_1_ states with a significant CT-excited state character in general^8,13^. In other words, the direct spin-flip between S_1_ and T_1_, which have the same CT-type molecular orbital (MO) configuration, should be inefficient as it is quantum-mechanically forbidden according to the conservation of total angular momentum^14,15^. This presents a conundrum, particularly regarding the occurrence of efficient spin-flip in these CT-type TADF molecules.

**Fig. 1:**
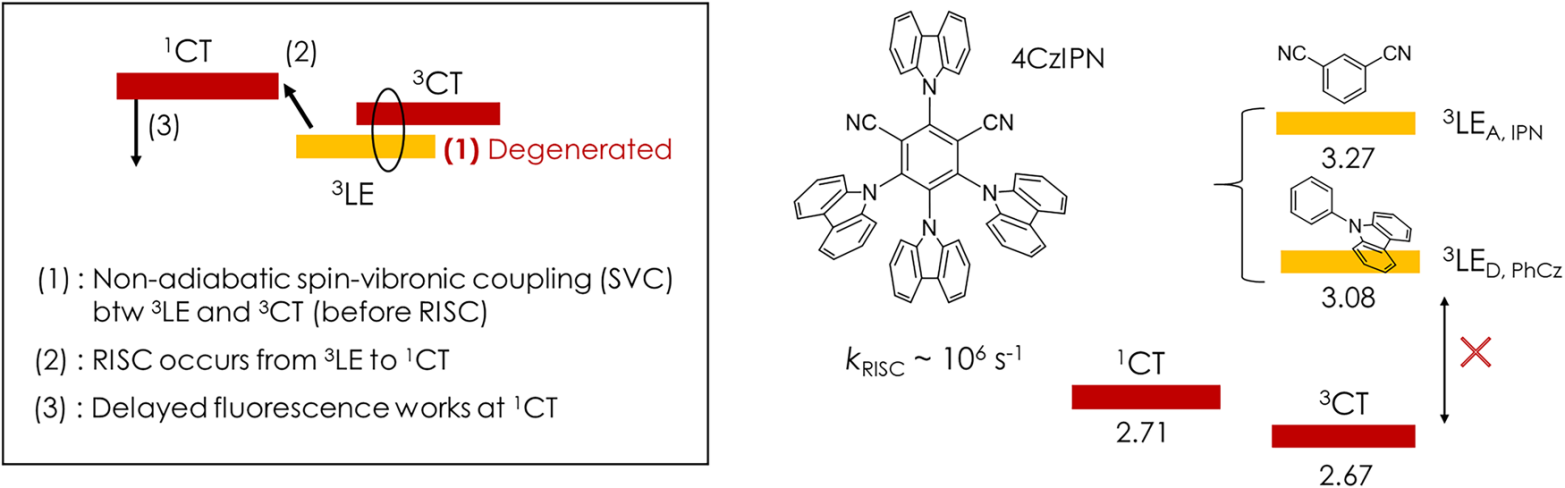
The necessity of generalization in spin-flip mechanism in CT-type clusters. The need for a generalized spin-flip mechanism and the illustration of counterexample, 4CzIPN molecule, comprising multi-donor Cz and acceptor of IPN moiety. The high energy of locally excited (LE) triplet states for IPN (^3^LE_A,IPN_) and PhCz (^3^LE_D,PhCz_) renders them unsuitable for effective interaction (SVC) with the low-lying CT state. Here, the superscript for CT denotes the spin multiplicity.

To induce spin-flipping in CT-type TADF molecular systems, the MO transition between two excited states with different spin multiplicities requires a torque enabling an electron’s spin flipping^16^. Therefore, it has been proposed that the presence of the locally excited triplet state (^3^LE) serves as an intermediate triplet state with a considerable change in MO. It contributes to rapid vibrational coupling known as spin-vibronic coupling (SVC), which results in the triplet equilibrium with a CT triplet state (^3^CT) and thus mediates the direct spin-orbit coupling (SOC) between CT states^17–20^. Among many notable efforts to generalize the spin interconversion process^21–23^, the use of electron spin resonance has shown that a strongly mixed CT/LE character driven by an available rotational or torsional freedom leads to a reversible spin-flip route^16,24^. In particular, donor-acceptor (D-A) or donor-acceptor-donor (D-A-D) type TADF molecular systems with this property have been shown to support efficient ISC between the singlet and triplet excited states (S_1_-T_1_). On the other hand, it was shown that a series of CT-type molecular systems do not conform to this mechanism^23^. The seminal TADF molecule, 1,2,3,5-tetrakis(carbazol-9-yl)-4,6-dicyanobenzene (4CzIPN)^1^, comprising a multiple of carbazole (Cz) donors and an acceptor of isophthalonitrile (IPN), can be an exemplary model system to study the generalized mechanisms of the spin-flip process in TADF molecules since their ^3^LE energies^21,23,25^ is rather too high to couple with low-lying CT state as summarized in Fig. 1. As stated in the previous report^23^, the spin-flip processes occur through a specific intermediate higher-lying triplet (T) state, which is associated with the electronic structure of a partial molecular framework within the CT-type molecule.

**Fig. 2:**
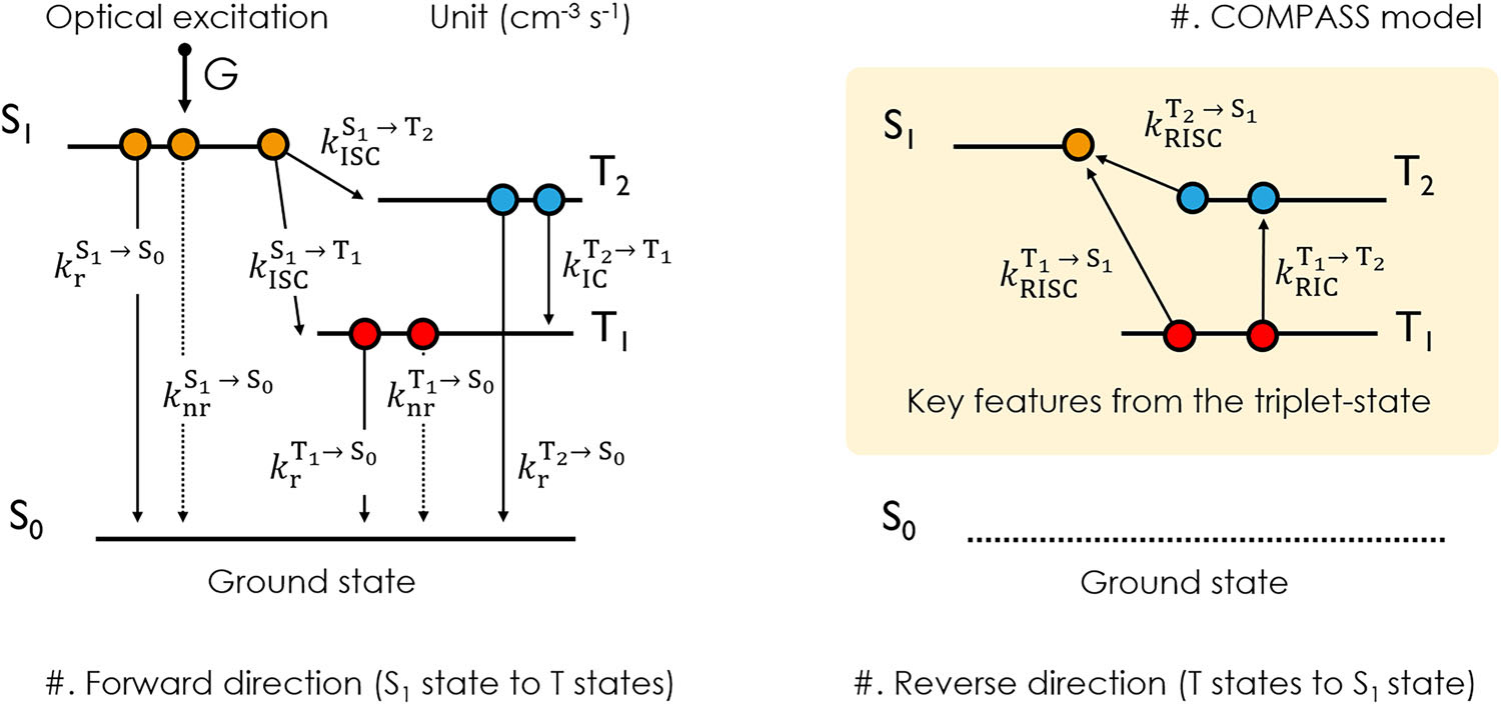
The schematic energy diagram of COMPASS model. The schematic energy diagram of COMPASS model after the optical pumping. Key rate constants governing electronic transitions in IPN derivatives; ${k}_{{{{{{\rm{r}}}}}}}^{{{{{{{\rm{S}}}}}}}_{1}\to {{{{{{\rm{S}}}}}}}_{0}}$ for radiative singlet decay at S_1_ state, ${k}_{{{{{{\rm{nr}}}}}}}^{{{{{{{\rm{S}}}}}}}_{1}\to {{{{{{\rm{S}}}}}}}_{0}}$ for non-radiative singlet decay at S_1_ state, ${k}_{{{{{{\rm{ISC}}}}}}}^{{{{{{{\rm{S}}}}}}}_{1}\to {{{{{{\rm{T}}}}}}}_{1}}$ for intersystem crossing (ISC) from S_1_ to T_1_ state, ${k}_{{{{{{\rm{ISC}}}}}}}^{{{{{{{\rm{S}}}}}}}_{1}\to {{{{{{\rm{T}}}}}}}_{2}}$ for ISC from S_1_ to T_2_ state, ${k}_{{{{{{\rm{r}}}}}}}^{{{{{{{\rm{T}}}}}}}_{1}\to {{{{{{\rm{S}}}}}}}_{0}}$ for radiative triplet decay at T_1_ state, ${k}_{{{{{{\rm{nr}}}}}}}^{{{{{{{\rm{T}}}}}}}_{1}\to {{{{{{\rm{S}}}}}}}_{0}}$ for the non-radiative triplet decay at T_1_ state, ${k}_{{{{{{\rm{RISC}}}}}}}^{{{{{{{\rm{T}}}}}}}_{1}\to {{{{{{\rm{S}}}}}}}_{1}}$ for reverse ISC (RISC) from T_1_ to S_1_ state, ${k}_{{{{{{\rm{RIC}}}}}}}^{{{{{{{\rm{T}}}}}}}_{1}\to {{{{{{\rm{T}}}}}}}_{2}}$ for reverse internal conversion (RIC) from T_1_ to T_2_ state, ${k}_{{{{{{\rm{r}}}}}}}^{{{{{{{\rm{T}}}}}}}_{2}\to {{{{{{\rm{S}}}}}}}_{0}}$ for radiative triplet decay at T_2_ state, ${k}_{{{{{{\rm{IC}}}}}}}^{{{{{{{\rm{T}}}}}}}_{2}\to {{{{{{\rm{T}}}}}}}_{1}}$ for IC from T_2_ to T_1_ state, ${k}_{{{{{{\rm{RISC}}}}}}}^{{{{{{{\rm{T}}}}}}}_{2}\to {{{{{{\rm{S}}}}}}}_{1}}$ for RISC from T_2_ to S_1_ state.

From a classical perspective, the kinetic description of independent particles (excitons) can be used to quantify the spin-flip rate in a TADF molecular system in the context of three electronic states: S_1_, T_1,_ and the ground state (S_0_)^9,26^. Additionally, under the framework of Fermi’s golden rule, the semi-classical prediction for the rate constant of reverse ISC (*k*_RISC_) can also be formulated by the introduction of Marcus theory^8,27^, which is limited to the high-temperature regime where the transition rate relies on the adiabatic energy difference between the two excited states (i.e., S_1_ ↔ T_1_ or S_1_ ↔ T_2_). Particularly, these computed spin-flip rates weighted from the multiple excited triplet states match well with experimentally obtained values from the rate equation of the three-level model^8^. This allows us to explain the spin-flip through direct SOC dependence on the difference of MO change if the potential energy surface (PES) for the excited states, e.g., S_1_, T_1_, and T_2_ states, do not give rise to a large coordinate change from its ground state^28^. This is, however, puzzling in that the three-level model inherently does not include the high-lying T states, such as a driving force T_2_ with a significant ^3^LE character. It is important to note that, while we can determine the initial and final electronic states involved in the spin-flip between S_1_ and T_1_ states, the exact dynamic processes and the role of intermediate high-lying T states are still not fully comprehended. Therefore, our aim is to establish a comprehensive analytical spin-flips (COMPASS) model that can reveal the intermediate steps involved in the dynamic processes and unify a framework that can describe a variety of CT-type TADF photophysical systems as depicted in Fig. 2.

## Results and discussions

### The implication of CT-type molecules with an energetically inverted triplet MO character

**Fig. 3:**
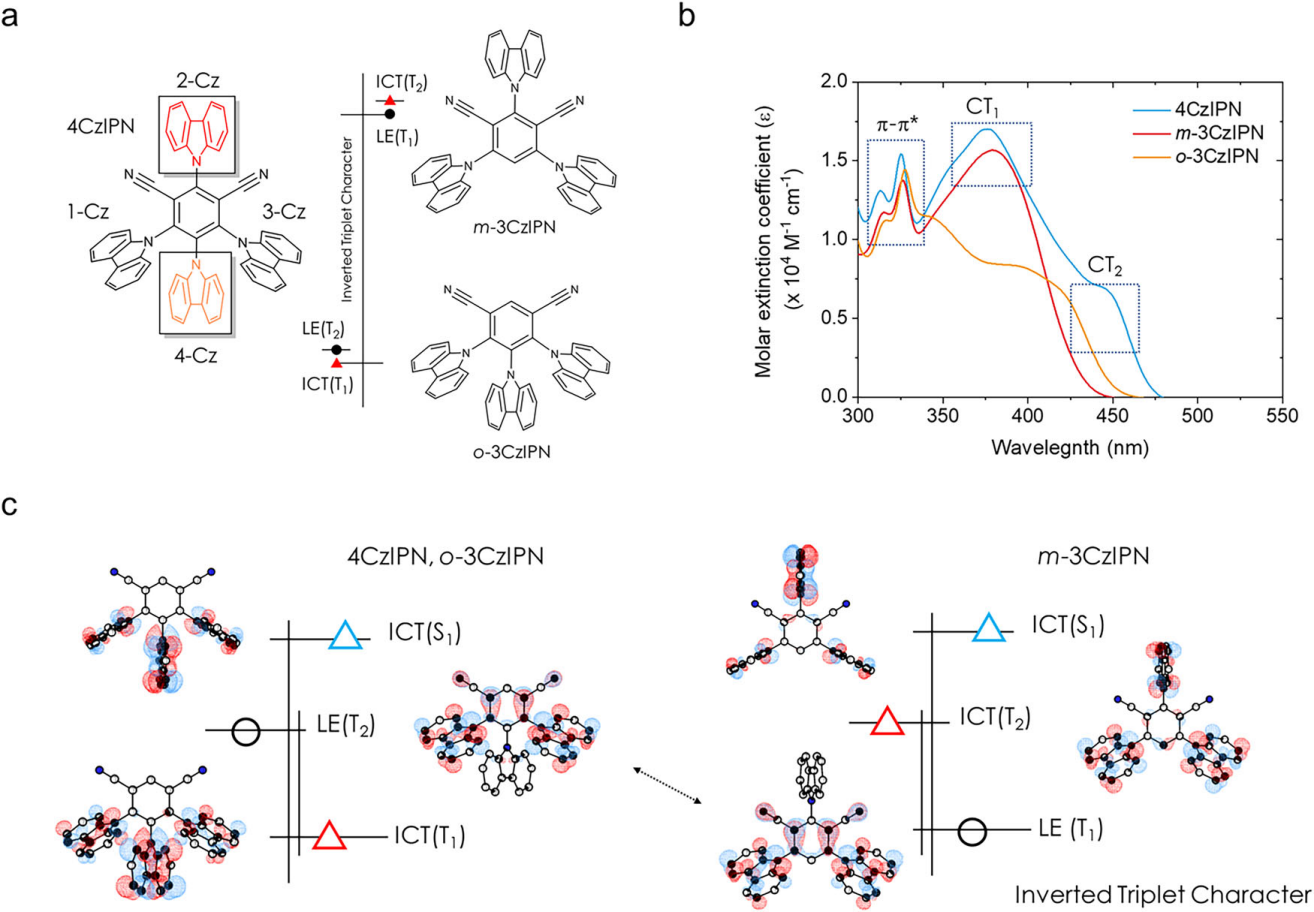
An outline of the underlying spin-flip process for IPN derivatives. **a** 4CzIPN molecule and its partial structure of IPN derivatives (*o*-3CzIPN and *m*-3CzIPN). Note that the triplet excitation character exhibits an inverted energetic ordering, with ^3^LE (T_2_ state) > ^3^ICT (T_1_ state) for both 4CzIPN and *o*-3CzIPN cases, while for *m*-3CzIPN, ^3^ICT (T_2_ state) > ^3^LE (T_1_ state). We hereby abbreviate the electronic state with a significant intramolecular charge-transfer-character as ICT (indicated by a red triangle) and LE (with black circle), specifically for a substantial amount of LE character. **b** Absorption spectra for the IPN derivatives (toluene, 0.05 mM). The dotted rectangle corresponds to the range of particular types of electronic transition states (i.e., π- π* or CT) for IPN-derivative. **c** The order in energy for IPN-derivative and natural transition orbital (NTOs) analyses (only hole wave function is depicted) at the optimized T_1_, T_2_, and S_1_ geometry of *o*-3CzIPN (left) and *m*-3CzIPN (right), using LC-w*PBE/6-31G** at TD-DFT within TDA. The red triangle signifies the dominant CT character in the triplet state, the blue triangle denotes the predominant CT character in S_1_ state, and the black circle represents the triplet state characterized by dominant LE character (refer to IPN site).

In this context, we revisited the significance of the high-lying T state in the spin-flip process of CT-type molecules, as the underlying mechanism governing the role of these states still remains unclear. We thus investigated the spin-flip processes for IPN-derivative, using the 4CzIPN molecule and its partial molecular structure detached to the Cz-unit, namely 4,5,6-tri(9*H*-carbazol-9-yl)isophthalonitrile (*o*-3CzIPN) and 2,4,6-tri(9*H*-carbazol-9-yl)isophthalonitrile (*m*-3CzIPN), so as to understand the pathways of the spin-flip with the eventual goal of achieving greater control over spin-flip rates (Fig. 3a). To this end, we first investigated the fundamental photophysical properties of the IPN sets, 4CzIPN, *o*-3CzIPN, and *m*-3CzIPN (see Supplementary Figs. 1–5 in Supplementary Information, SI). Figure 3b presents the absorption spectra of 4CzIPN and its partial molecules, corresponding to π–π* band (i.e., the LE state of the Cz moieties) around 325–328 nm for all species, the localized intramolecular CT (^lo^ICT, CT_1_) state at the main peak around 376–379 nm for both 4CzIPN and *m*-3CzIPN, and the delocalized intramolecular CT (^de^ICT, CT_2_) state with the long-tail absorption shoulder peak observed after approximately 417 and 447 nm for *o*-3CzIPN and 4CzIPN, respectively. Note that the electronic structures of 4CzIPN still remain persevered in its partial molecules, exhibiting two types of CT characters, ^lo^CT (CT_1_) and ^de^CT (CT_2_), in their respective partial structure^21,23^.

The preserved electronic resemblance to the 4CzIPN molecule in the absorption spectra suggests that its partial backbone, *o*-3CzIPN, and *m*-3CzIPN, have the potential to possess the high-lying T states that play a critical role in facilitating efficient spin-flip processes in CT-type TADF molecular systems^23^. To strengthen the experimental evidence, we conducted both steady-state room-temperature photoluminescence (RTPL) and low-temperature PL (LTPL) spectroscopy on IPN derivatives, as shown in Supplementary Figs. 1–3. All IPN-derivative exhibit a predominant CT character at the S_1_ state (^1^CT), with a featureless broad emissive property. The LTPL response of the 4CzIPN molecule differs from that of the other components, as the phosphorescence spectrum shows a clear tendency to split into two main transitions in toluene for *o*-3CzIPN and *m*-3CzIPN cases. This unique electronic transition, which is not due to vibrational transitions, disappears when the partial molecules, *m*-3CzIPN and *o*-3CzIPN, are doped onto an *m*CBP host film, resulting in broadened emission spectra. This indicates that the presence of a decoupled high-lying T state (i.e., T_2_ state) coexists with the lowest triplet state due to the lowered internal conversion (IC) rate between the T_1_ and T_2_ states. This will be delineated later.

To understand the nature of electronic structures for IPN sets, we conducted quantum-chemical calculations (see ‘Computational details’ in SI and Supplementary Figs. 6–11 for more details). Especially, in the T_1_ and T_2_ equilibrated geometries for the *o*-3CzIPN and *m*-3CzIPN molecules, they exhibited a common MO excitation shape, but their energy ordering of the MO character was completely inverted. In particular, the hole density is strongly localized on the 1,3-position Cz and IPN units (refer to Fig. 3a, c). The common MO feature is situated on 4,6-di(9*H*-carbazol-9-yl)isophthalonitrile (*m*-2CzIPN) molecular backbone in the T_1_ state for *m*-3CzIPN and T_2_ state for *o*-3CzIPN, respectively. Note that the energy alignment is reversed for the hole wave function in the case of *m*-3CzIPN (see also Fig. 3c and Supplementary Figs. 7–9). Here, we can also find this specific MO triplet excitation is employed at the vertical T_2_ transition referenced from the T_1_ geometry of 4CzIPN as shown in Supplementary Fig. 7. Therefore, we can conclude that a specific triplet state with a common excitation MO shape represents the electronic structure of a partial molecular framework within the 4CzIPN molecule.

In this regard, we classified the system based on the energy alignment; 4CzIPN and *o*-3CzIPN follow the same energetic order of ^1^CT (S_1_) > ^3^LE (T_2_) > ^3^CT (T_1_), while *m*-3CzIPN shows an inverted triplet character with the order of ^1^CT (S_1_) > ^3^CT (T_2_) > ^3^LE (T_1_) as shown in Fig. 3c. The optimized S_1_ and T_1_ states of 4CzIPN are of particular interest because they exhibit only a minor contribution from the 2-Cz unit to the overall hole density distribution in each state (see Supplementary Fig. 7). Given that the extensive discussions for a series of natural transition orbital (NTO) analyses depicted in ‘Computational details’ in SI, thus, the contribution from the 2-position Cz unit to hole wave function can be a driving force in enabling a large MO difference in the context of the conservation of total angular momentum. This MO change could lead to a more efficient ISC or RISC process in the system. The available rotational freedom of the 2-position Cz unit in this IPN archetype (i.e., 4CzIPN) would also contribute to a MO change (refer to Supplementary Figs. 10 and 11), being consistent with the previous reports^12,29,30^. The extent of overlap between the hole and electron distributions of S_1_ and T_1_ shows a similar propensity, indicating that the strength of CT character can be increased with the clockwise dihedral change of the 2-Cz moiety^23^. However, there is no change observed in the T_2_ state, which has an identical MO excitation character (i.e., a hole/particle density distribution in *o*-3CzIPN at the T_2_ state and *m*-3CzIPN at the T_1_ state). This leads to an enhanced SOC matrix element (SOCME) that would enable a more efficient ISC/RISC between the S_1_ and T_2_ state, as opposed to the S_1_ and T_1_ state for the 4CzIPN case (see also Supplementary Fig. 11).

To substantiate our assertion, we extend our theoretical modeling to condensed solid films on the basis of molecular dynamics, particularly for a 5.0 wt.% doped *m*CBP film (refer to ‘Computational details’ in Methods and Supplementary Figs. 12–18 in SI for more information). The cases for 4CzIPN, *o*-3CzIPN, and *m*-3CzIPN, are modeled and described as a form of the electronic structure distribution, captured at the snapshot from the converged frame (at 100.0 ns). Remarkably, our findings demonstrate that when the electronic structure distributions for T_1_ and T_2_ states, obtained from individual molecule at the converged frame, are statistically treated as a system (designated as T_Cluster_), they can effectively mimic the LTPL responses observed in the film state (refer to Supplementary Figs. 1–3 for experimental data and Supplementary Figs. 13–15 for theoretical models). As a result, we can offer the theoretical evidence that the presence of two distinct electronic transitions, namely T_1_ and T_2_ states, in solution-state, particularly in the cases of *o*-3CzIPN and *m*-3CzIPN. It is also crucial to note that the similarity in MO excitation characters between T_1_ and T_2_ states is associated with the IC rate (*k*_IC_), especially when compared to the case of 4CzIPN.

At first glance, our observation seems to challenge Kasha’s rule^31,32^, which posits that photon emission primarily occurs from the most stable excited state of a given spin multiplicity. Although there is some ambiguity in the physical interpretation of this result, prior spectroscopic studies^33–35^ done for the 4CzIPN molecule strongly corroborate our experimental evidence, suggesting the potential co-existence of a high-lying T state at low temperatures. Considering the assigned energy order of the IPN-derivative depicted in Fig. 3c, one can see that the disparity in SOCME distribution, obtained for 4CzIPN, *o*-3CzIPN, and *m*-3CzIPN with their respective condensed-film state model (Supplementary Fig. 18), primarily arises from a specific triplet state sharing a common excitation MO shape. Their ^3^LE states are deemed to play a critical role in generating a large SOCME, facilitating an efficient spin-flip process. Intriguingly, the efficient ISC route of *m*-3CzIPN appears to deviate from that of the 4CzIPN and *o*-3CzIPN, particularly in the context of inverted energy levels.

### The COMPASS model applicable to a wide variety of CT-type molecules

Given that these IPN derivatives exhibit almost similar *k*_RISC_ in the range of ~ 10^6 ^s^−1^ in the solid-state, as experimentally determined under the three-level model, taking into account the S_1_, T_1_, and S_0_ states (refer to Supplementary Figs. 4 and 5 and Supplementary Tables 1–4), it is justified that a comprehensive dynamic model is necessary to achieve a complete understanding of spin-flip processes. This includes the consideration of high-lying T states, such as the T_2_ state, in these molecular systems. We extended the model, incorporating temperature dependence, to a universal kinetic model, known as the three-level model, involving S_1_, T_1_, and S_0_ states^9,26^. This model effectively described the delayed component (*k*_d_) for a diverse range of CT-type molecules. It can be asserted that if the system adheres to this model, there is no need for the development of an alternative one. In pursuit of this, we explored two possible variants of the three-level models – *System A* (consisting of T_1_, T_2_, and S_0_ states) and *System B* (comprising S_1_, T_2_, and S_0_ states) – capable of explaining exciton decays obtained from the transient PL (Tr-PL) profiles over a wide range of temperature (refer to “Exciton Dynamics” section in SI.).

**Fig. 4:**
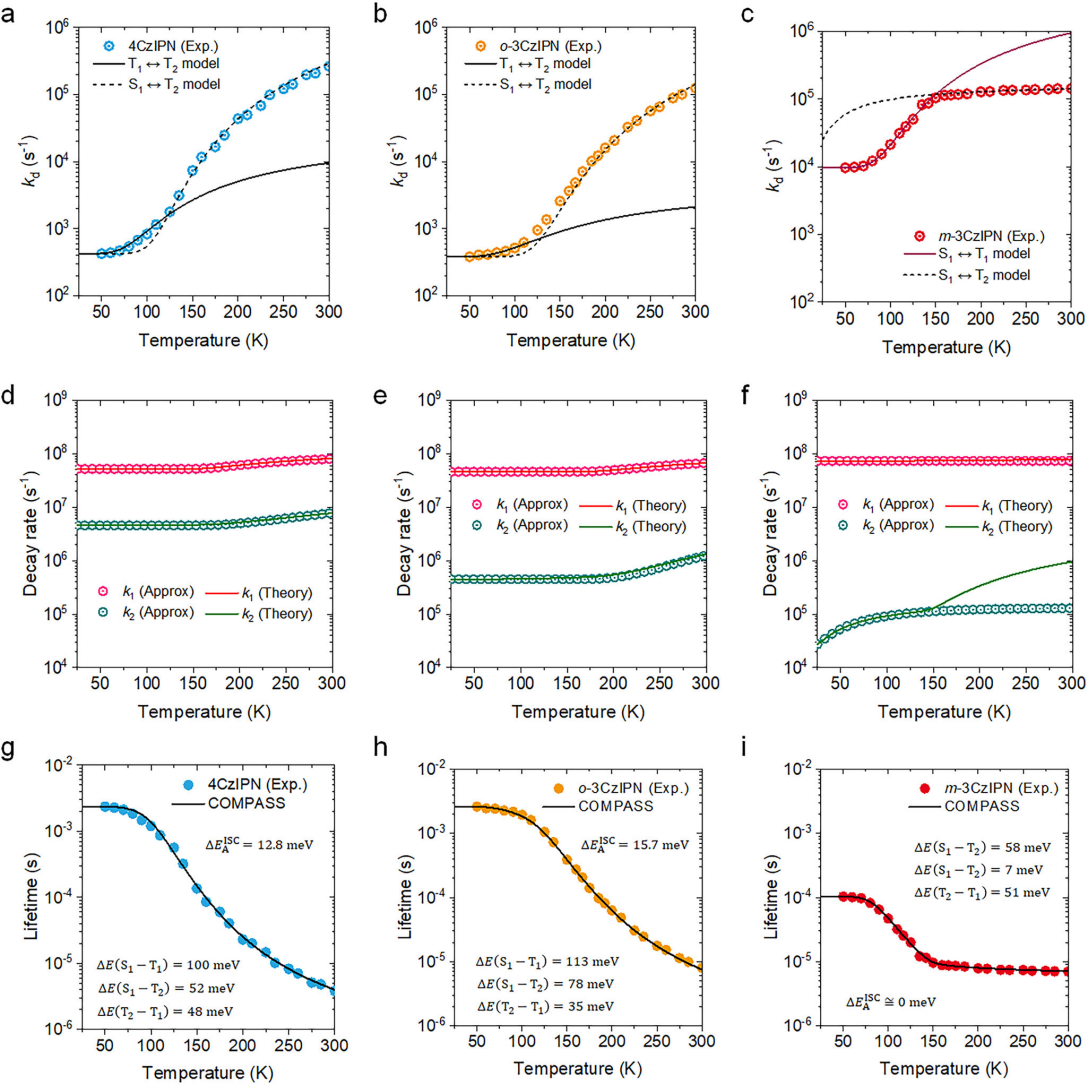
Temperature dependence of decay rate response based on COMPASS model. The temperature (*T*) dependence of the delayed component (*k*_d_) responses for (**a**) 4CzIPN, (**b**) *o*−3CzIPN, and (**c**) *m*−3CzIPN by using a series of three-level models. Refer to Eqs. (S3), (S5), and (S7) in SI for S_1_ ↔ T_1_ (the three-level), T_1_ ↔ T_2_ (*System A*), and S_1_ ↔ T_2_ (*System B*) model, respectively. The decay rate responses for *k*_1_ (Theory) and *k*_2_ (Theory) for (**d**) 4CzIPN, (**e**) *o*-3CzIPN, and (**f**) *m*-3CzIPN molecular system on the basis of COMPASS model. Hereby, we approximated a couple of rate decays, ${k}_{{{{{{\rm{n}}}}}}}({{{{{\rm{Approx.}}}}}})$, as follows: ${k}_{1}({{{{{\rm{Approx.}}}}}})\cong {k}_{{{{{{\rm{r}}}}}}}^{{{{{{{\rm{S}}}}}}}_{1}\to {{{{{{\rm{S}}}}}}}_{0}}+{k}_{{{{{{\rm{nr}}}}}}}^{{{{{{{\rm{S}}}}}}}_{1}\to {{{{{{\rm{S}}}}}}}_{0}}+{k}_{{{{{{\rm{ISC}}}}}}}^{{{{{{{\rm{S}}}}}}}_{1}\to {{{{{{\rm{T}}}}}}}_{2}}+{k}_{{{{{{\rm{ISC}}}}}}}^{{{{{{{\rm{S}}}}}}}_{1}\to {{{{{{\rm{T}}}}}}}_{1}}$ and ${k}_{2}({{{{{\rm{Approx.}}}}}})\cong {k}_{r}^{{{{{{{\rm{T}}}}}}}_{2}\to {{{{{{\rm{S}}}}}}}_{0}}+{k}_{{{{{{\rm{IC}}}}}}}^{{{{{{{\rm{T}}}}}}}_{2}\to {{{{{{\rm{T}}}}}}}_{1}}+{P}_{2}{k}_{{{{{{\rm{RISC}}}}}}}^{{{{{{{\rm{T}}}}}}}_{2}\to {{{{{{\rm{S}}}}}}}_{1}}$, respectively. The experimental response of delayed lifetime in the range of temperature for IPN-species; (**g**) 4CzIPN, (**h**) *o*-3CzIPN, and (**i**) *m*-3CzIPN molecule on *m*CBP host film (conc. 5.0 wt.% doped).

A set of models comprising only three electronic states proved inadequate in fully explaining the experimental PL decay responses for the solid-state films of these CT-type TADF molecules. However, we did observe a distinct response in the delayed component (*k*_d_) of the experimental PL at a specific temperature, indicating a change in the involvement of electronic states, as illustrated in Fig. 4a–c. Our investigation revealed that, for 4CzIPN (Fig. 4a) and *o*-3CzIPN (Fig. 4b), the *k*_d_ response at low temperature was consistent with *System A*, where the T_1_ and T_2_ states were involved. However, as the temperature increased, the experimental *k*_d_ response deviated from *System A* and instead followed that of *System B* (i.e., S_1_ ↔ T_2_ interaction). Additionally, we found that the behavior of *k*_d_ for *m*-3CzIPN (Fig. 4c) at low temperatures adhered to the three-level model involving S_1_ ↔ T_1_ interaction, deviating from *System A*. However, at high temperatures, the *k*_d_ response followed that of *System B*, akin to those of 4CzIPN and *o*-3CzIPN cases.

Based on these observations, we can conjecture that the presence of an energy-wise inverted triplet state with a common excitation MO shape at the T_1_ state of *m*-3CzIPN could account for the disparity in the *k*_d_ response observed in 4CzIPN and *o*-3CzIPN at low temperatures. Supplementary Figs. 19a–c shows the temperature dependence of ISC for *m*-3CzIPN, which differs from the others as it does not have an activation energy of forward ISC (${\Delta E}_{{{{{{\rm{A}}}}}}}^{{{{{{\rm{ISC}}}}}}}$). In particular, the ISC process in *m*-3CzIPN does not involve an uphill process (i.e., ${\Delta E}_{{{{{{\rm{A}}}}}}}^{{{{{{\rm{ISC}}}}}}}$ = 0 meV). This can be attributed to the presence of an energetically inverted ^3^LE character at the T_1_ state, which plays a crucial role in the spin conversion of the molecular system. This state not only makes an inflection point (or the participation of new electronic state) in the *k*_d_ response, indicating its involvement in inducing the difference in the electronic MO contribution that gives rise to the spin-flip process, but is also associated with its energetic order in triplet manifolds.

In this regard, to fully account for the observed experimental responses, we propose a comprehensive analytical spin-flips (COMPASS) model that incorporates a four-level structure: the S_1_, T_2_, T_1_, and S_0_ states. To delve into the system (refer to Fig. 2), we can describe the exciton dynamics within the classical rate equation framework as follows:

1 $${d{{{{{\rm{S}}}}}}}_{1}\left(t\right)/{dt}=-A{{{{{{\rm{S}}}}}}}_{1}\left(t\right)+{k}_{{{{{{\rm{RISC}}}}}}}^{{{{{{{\rm{T}}}}}}}_{2}\to {{{{{{\rm{S}}}}}}}_{1}}{{{{{{\rm{T}}}}}}}_{2}\left(t\right)+{k}_{{{{{{\rm{RISC}}}}}}}^{{{{{{{\rm{T}}}}}}}_{1}\to {{{{{{\rm{S}}}}}}}_{1}}{{{{{{\rm{T}}}}}}}_{1}\left(t\right)+G$$

2 $${d{{{{{\rm{T}}}}}}}_{2}\left(t\right)/{dt}={k}_{{{{{{\rm{ISC}}}}}}}^{{{{{{{\rm{S}}}}}}}_{1}\to {{{{{{\rm{T}}}}}}}_{2}}{{{{{{\rm{S}}}}}}}_{1}\left(t\right)-B{{{{{{\rm{T}}}}}}}_{2}\left(t\right)+{k}_{{{{{{\rm{RIC}}}}}}}^{{{{{{{\rm{T}}}}}}}_{1}\to {{{{{{\rm{T}}}}}}}_{2}}{{{{{{\rm{T}}}}}}}_{1}\left(t\right)$$

3 $${d{{{{{\rm{T}}}}}}}_{1}\left(t\right)/{dt}={k}_{{{{{{\rm{ISC}}}}}}}^{{{{{{{\rm{S}}}}}}}_{1}\to {{{{{{\rm{T}}}}}}}_{1}}{{{{{{\rm{S}}}}}}}_{1}\left(t\right)+{k}_{{{{{{\rm{IC}}}}}}}^{{{{{{{\rm{T}}}}}}}_{2}\to {{{{{{\rm{T}}}}}}}_{1}}{{{{{{\rm{T}}}}}}}_{2}\left(t\right)-C{{{{{{\rm{T}}}}}}}_{1}\left(t\right)$$

Here, *A*, *B*, and *C* correspond to the sum of total exciton consumption routes at S_1_, T_2_, and T_1_ states, respectively. *G* is the singlet exciton generation ratio by optical pumping. ${k}_{{{{{{\rm{ISC}}}}}}}^{{{{{{{\rm{S}}}}}}}_{1}\to {{{{{{\rm{T}}}}}}}_{{{{{{\rm{n}}}}}}}}$ denote the ISC pathway from S_1_ to T_n_ state, where the subscript *n* can have values 1 to 2, reflecting the accessible triplet states. Similarly, ${k}_{{{{{{\rm{RISC}}}}}}}^{{{{{{{\rm{T}}}}}}}_{{{{{{\rm{n}}}}}}}\to {{{{{{\rm{S}}}}}}}_{1}}$ is designated as the RISC process from T_n_ to S_1_ state, respectively.

As aforementioned, Fig. 4a–c confirmed a temperature-dependent change in the involvement of electronic states at a specific temperature (*T*). We thus can establish the connection between these changes and the population of electronic states, T_1_ and T_2_ states, by adopting the Boltzmann distribution. (*vide infra*)

4 $${k}_{{{{{{\rm{RIC}}}}}}}^{{{{{{{\rm{T}}}}}}}_{1}\to {{{{{{\rm{T}}}}}}}_{2}}(T)={k}_{{{{{{\rm{IC}}}}}}}^{{{{{{{\rm{T}}}}}}}_{2}\to {{{{{{\rm{T}}}}}}}_{1}}{e}^{-\frac{\Delta {{{{{{\rm{E}}}}}}}_{{{{{{{\rm{T}}}}}}}_{2}-{{{{{{\rm{T}}}}}}}_{1}}}{{k}_{{{{{{\rm{B}}}}}}}T}}$$

In this work, ${k}_{{{{{{\rm{IC}}}}}}}^{{{{{{{\rm{T}}}}}}}_{2}\to {{{{{{\rm{T}}}}}}}_{1}}$ corresponds to the IC route from T_2_ to T_1_ state, and ${k}_{{{{{{\rm{RIC}}}}}}}^{{{{{{{\rm{T}}}}}}}_{1}\to {{{{{{\rm{T}}}}}}}_{2}}$ represents the reverse IC (RIC) process from T_1_ to T_2_ state. More detailed information on COMPASS model is summarized in the “Exciton Dynamics” section of SI. The COMPASS model provides three characteristic decay rates *k*_1_, *k*_2_, and *k*_3_, and the time evolution (*t*) for a pair of excited states (S_1_, T_2_, and T_1_ states) can be described by a sum of three-exponential decays, ${\sum }_{n=1}^{3}{O}_{n}{e}^{-{k}_{n}t}$ where ${O}_{n}$ is determined by an initial condition. As an exemplary model, *S*_1_(*t*), directly associated with PL intensity, can be expressed as follows:

5 $${{{{{{\rm{S}}}}}}}_{1}\left(t\right)=	\frac{\left(B-{k}_{1}\right)\left(C-{k}_{1}\right)-{k}_{{{{{{\rm{RIC}}}}}}}^{{{{{{{\rm{T}}}}}}}_{1}\to {{{{{{\rm{T}}}}}}}_{2}}{k}_{{{{{{\rm{IC}}}}}}}^{{{{{{{\rm{T}}}}}}}_{2}\to {{{{{{\rm{T}}}}}}}_{1}}}{\left({k}_{2}-{k}_{1}\right)\left({k}_{3}-{k}_{1}\right)}{e}^{-{k}_{1}t} \\ 	+ \frac{\left(B-{k}_{2}\right)\left(C-{k}_{2}\right)-{k}_{{{{{{\rm{RIC}}}}}}}^{{{{{{{\rm{T}}}}}}}_{1}\to {{{{{{\rm{T}}}}}}}_{2}}{k}_{{{{{{\rm{IC}}}}}}}^{{{{{{{\rm{T}}}}}}}_{2}\to {{{{{{\rm{T}}}}}}}_{1}}}{({k}_{1}-{k}_{2})({k}_{3}-{k}_{2})}{e}^{-{k}_{2}t} \\ 	+ \frac{\left(B-{k}_{3}\right)\left(C-{k}_{3}\right)-{k}_{{{{{{\rm{RIC}}}}}}}^{{{{{{{\rm{T}}}}}}}_{1}\to {{{{{{\rm{T}}}}}}}_{2}}{k}_{{{{{{\rm{IC}}}}}}}^{{{{{{{\rm{T}}}}}}}_{2}\to {{{{{{\rm{T}}}}}}}_{1}}}{({k}_{1}-{k}_{3})({k}_{2}-{k}_{3})}{e}^{-{k}_{3}t}$$

### The origin of spin-flip route for CT-type molecule

It is worth noting that the value of *k*_1_ value [*k*_1_ (Theory)] obtained from COMPASS model aligns well with the prompt region (nanoseconds scale) of the experimental Tr-PL data in all cases, encompassing processes occurring in the S_1_ state (see Fig. 4d–f). This is supported by the consistency of the approximated rate decays for *k*_1_ (Theory); *k*_1_ (Approx.) = ${k}_{{{{{{\rm{r}}}}}}}^{{{{{{{\rm{S}}}}}}}_{1}\to {{{{{{\rm{S}}}}}}}_{0}}+{k}_{{{{{{\rm{nr}}}}}}}^{{{{{{{\rm{S}}}}}}}_{1}\to {{{{{{\rm{S}}}}}}}_{0}}+{k}_{{{{{{\rm{ISC}}}}}}}^{{{{{{{\rm{S}}}}}}}_{1}\to {{{{{{\rm{T}}}}}}}_{2}}+{k}_{{{{{{\rm{ISC}}}}}}}^{{{{{{{\rm{S}}}}}}}_{1}\to {{{{{{\rm{T}}}}}}}_{1}}$. In addition, we have identified that *k*_2_ [*k*_2_ (Thoery)] as an important parameter capturing the high-lying T_2_ process, as supported by the consistency of an approximated rate model for *k*_2_, i.e., *k*_2_ (Approx.) = ${k}_{r}^{{{{{{{\rm{T}}}}}}}_{2}\to {{{{{{\rm{S}}}}}}}_{0}}+{k}_{{{{{{\rm{IC}}}}}}}^{{{{{{{\rm{T}}}}}}}_{2}\to {{{{{{\rm{T}}}}}}}_{1}}+{P}_{2}{k}_{{{{{{\rm{RISC}}}}}}}^{{{{{{{\rm{T}}}}}}}_{2}\to {{{{{{\rm{S}}}}}}}_{1}}$. The detailed description of the corresponding physical values is provided in the caption of Fig. 2). Therefore, with increasing temperature, there is an increment of *k*_1_ and *k*_2_ in the activation of ISC from S_1_ to T_2_ for 4CzIPN (Fig. 4d) and o-3CzIPN (Fig. 4e). This follows the energy order of ^1^CT (S_1_), ^3^LE (T_2_), and ^3^CT (T_1_), leading to an efficient RISC from T_2_ to S_1_ (${k}_{{{{{{\rm{RISC}}}}}}}^{{{{{{{\rm{T}}}}}}}_{2}\to {{{{{{\rm{S}}}}}}}_{1}}$) (refer to the hole density distribution in Fig. 3c).

It is important to note that *k*_2_ (Approx.) for *m*-3CzIPN case (Fig. 4i) deviated from that of the COMPASS model [i.e., *k*_2_ (Theory), solid line] in the vicinity of 150 *K*. This deviation marks an inflection point where the delayed lifetime response is constrained due to the efficient participation of RISC from T_1_ to S_1_, even at higher temperature (refer to Fig. 4c, i). Specifically, this indicates that ${k}_{{{{{{\rm{RISC}}}}}}}^{{{{{{{\rm{T}}}}}}}_{2}\to {{{{{{\rm{S}}}}}}}_{1}}$ (blue solid line) in *m*-3CzIPN is less likely than ${k}_{{{{{{\rm{RISC}}}}}}}^{{{{{{{\rm{T}}}}}}}_{1}\to {{{{{{\rm{S}}}}}}}_{1}}$ (red solid line) at high-temperature regime (over 200 *K* as calculated by COMPASS model in Supplementary Fig. 20) due to the sufficient thermal energy to induce direct spin-flip between the S_1_ and T_1_ states. While we can describe the involvement of an electronic state at 150 *K* using the three-level model (*System B*, in Fig. 4c), it is evident that a proper explanation of the spin dynamics is not achievable for *m*-3CzIPN case (Fig. 4i). In other words, a significant SOCME from ^3^LE character (central to IPN part) at the T_1_ state, rather than the T_2_ state with ^3^CT property, plays a critical role in the spin-conversion process (see Fig. 3c). This is consistent with our finding that an inverted triplet character in *m*-3CzIPN, a partial electronic structure of 4CzIPN, can be a key clue to revealing a major spin-flip route in these systems. Furthermore, within the framework of Fermi’s golden rule, we can arrive at the same conclusion by examining the theoretically predicted *k*_RISC_ values at the T_1_ and T_2_ states. Moreover, *m*-3CzIPN shows T_2_ state with a small SOCME (i.e., the interaction between pure CT characters), compared to others (4CzIPN and *o*-3CzIPN cases), as shown in Supplementary Fig. 21.

**Fig. 5:**
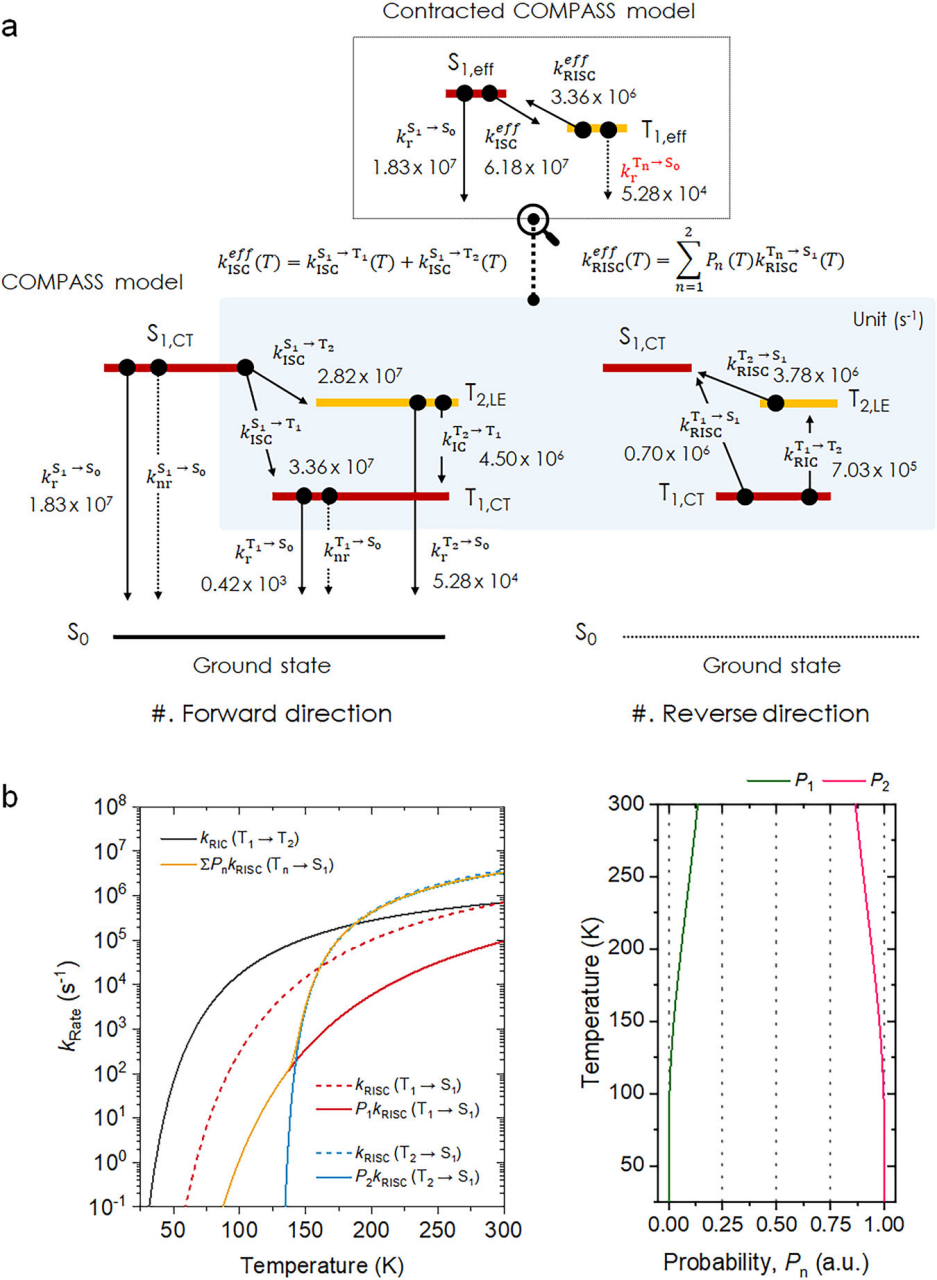
The full rate description for 4CzIPN case based on COMPASS model. **a** Proposed rate relations for 4CzIPN case at respective electronic states (i.e., S_1_, T_1_, T_2_, and S_0_ states) on the basis of COMPASS model. A simplified kinetic model (contracted COMPASS model) is derived from the COMPASS model, precisely conforming to the three-level model (refer to Supplementary Tables 4 and 5). **b** The rate constants (i.e., ${k}_{{{{{{\rm{RIC}}}}}}}^{{{{{{{\rm{T}}}}}}}_{1}\to {{{{{{\rm{T}}}}}}}_{2}}$, ${k}_{{{{{{\rm{RISC}}}}}}}^{{{{{{{\rm{T}}}}}}}_{1}\to {{{{{{\rm{S}}}}}}}_{1}}$, ${k}_{{{{{{\rm{RISC}}}}}}}^{{{{{{{\rm{T}}}}}}}_{2}\to {{{{{{\rm{S}}}}}}}_{1}}$, and ${k}_{{{{{{\rm{RISC}}}}}}}^{{{{{{\rm{eff}}}}}}}$) for 4CzIPN molecule (left) and the probability (${P}_{n}$) of the system occupying the electronic-state *n* in accordance with the temperature (right).

The dependence of delayed lifetime on temperatures, in accordance with the reciprocal of *k*_3_ value from COMPASS model, exhibits a good correspondence with the experimental results for all samples (see Fig. 4g–i). While the delayed lifetimes for all samples converge to a similar value at 300 *K*, their behaviors diverge significantly from one another with changing temperature (compare Fig. 4g–h with Fig. 4i). One might then wonder how COMPASS model generalizes the three-level model (Fig. 5a). To assess whether using the three-level model contracted from the COMPASS model is suitable, we introduced the effective rate constant of ISC (${k}_{{{{{{\rm{ISC}}}}}}}^{{eff}}$) and RISC (${k}_{{{{{{\rm{RISC}}}}}}}^{{eff}}$), by simplifying COMPASS model to the three-level, comprising a single effective singlet (S_1,eff_), triplet (T_1,eff_), and S_0_ states. *viz*,

6 $${k}_{{{{{{\rm{ISC}}}}}}}^{{{{{{\rm{eff}}}}}}}(T)={k}_{{{{{{\rm{ISC}}}}}}}^{{{{{{{\rm{S}}}}}}}_{1}\to {{{{{{\rm{T}}}}}}}_{1}}(T)+{k}_{{{{{{\rm{ISC}}}}}}}^{{{{{{{\rm{S}}}}}}}_{1}\to {{{{{{\rm{T}}}}}}}_{2}}(T)$$

7 $${k}_{{{{{{\rm{RISC}}}}}}}^{{{{{{\rm{eff}}}}}}}(T)=\mathop{\sum }\limits_{n=1}^{2}{P}_{n}(T){k}_{{{{{{\rm{RISC}}}}}}}^{{{{{{{\rm{T}}}}}}}_{{{{{{\rm{n}}}}}}}\to {{{{{{\rm{S}}}}}}}_{1}}(T)$$

The probability (${P}_{n}$) of the system occupying the electronic state *n* is defined by ${P}_{n}(T)=\frac{1}{Z}{e}^{-\beta {E}_{n}}$, where *Z* denotes the canonical partition function and ${e}^{-\beta {E}_{n}}$ is known as the Boltzmann factor. Here, *β* is the thermodynamic beta, defined as $\frac{1}{{k}_{{{{{{\rm{B}}}}}}}T}$. As an exemplary model, the P_1_ and P_2_ curves for 4CzIPN case dependent on the temperature is shown in Fig. 5b (right). The calculated results for *m*-3CzIPN and *o*-3CzIPN are summarized in Supplementary Figs. 20 and 22, respectively. Figure 5a shows the full rate relation at the respective electronic state for 4CzIPN case at 300 *K*. It is important to note that the value of ${k}_{{{{{{\rm{RISC}}}}}}}^{{{{{{{\rm{T}}}}}}}_{1}\to {{{{{{\rm{S}}}}}}}_{1}}$ with 0.70 × 10^6 ^s^−1^ is slower than ${k}_{{{{{{\rm{RISC}}}}}}}^{{{{{{{\rm{T}}}}}}}_{2}\to {{{{{{\rm{S}}}}}}}_{1}}$ with 3.78 × 10^6 ^s^−1^, strongly supporting the importance of the energy order of the system as discussed. It is noteworthy that in the case of *o*-3CzIPN, the value of ${k}_{{{{{{\rm{RISC}}}}}}}^{{{{{{{\rm{T}}}}}}}_{1}\to {{{{{{\rm{S}}}}}}}_{1}}$ at 0.34 × 10^6 ^s^−1^ is slower than ${k}_{{{{{{\rm{RISC}}}}}}}^{{{{{{{\rm{T}}}}}}}_{2}\to {{{{{{\rm{S}}}}}}}_{1}}$ at 1.02 × 10^6 ^s^−1^, akin to 4CzIPN case. However, for *m*-3CzIPN case, the value of ${k}_{{{{{{\rm{RISC}}}}}}}^{{{{{{{\rm{T}}}}}}}_{1}\to {{{{{{\rm{S}}}}}}}_{1}}$ at 6.60 × 10^6 ^s^−1^ is much more efficient than that of ${k}_{{{{{{\rm{RISC}}}}}}}^{{{{{{{\rm{T}}}}}}}_{2}\to {{{{{{\rm{S}}}}}}}_{1}}$ at 0.12 × 10^6 ^s^−1^, as obtained from COMPASS model (refer to Supplementary Tables 5 and 6).

We have intriguingly confirmed that the series of effective (eff.) rate constants for ISC/RISC match well with those determined by the three-level model (see Supplementary Table 4). For instance, *o*-3CzIPN exhibits the value of ${k}_{{{{{{\rm{RISC}}}}}}}^{{{{{{\rm{eff}}}}}}}$ with 0.88 × 10^6 ^s^−1^, and *m*-3CzIPN exhibits the value of ${k}_{{{{{{\rm{RISC}}}}}}}^{{{{{{\rm{eff}}}}}}}$ with 0.91 × 10^6 ^s^−1^, demonstrating complete consistency with the three-level model. As presented in Fig. 5b (left), we extended this approach to encompass for the entire temperature range. With this, we can dissect the rate relations at each individual temperature, particularly for triplet states such RIC, a set of RISCs, and effective RISC. In conclusion, this observation rationalizes the generalized form of the CT-type TADF molecular system through the COMPASS model (Fig. 5a). Repeatedly, we once again note that ${k}_{{{{{{\rm{RISC}}}}}}}^{{{{{{\rm{eff}}}}}}}$ for *o*-3CzIPN and *m*-3CzIPN reaches within a similar rate range at 300 *K*. This similarity implies that, by relying solely on the three-level model, we are unable to provide insight into the underlying spin-flip mechanism.

**Fig. 6:**
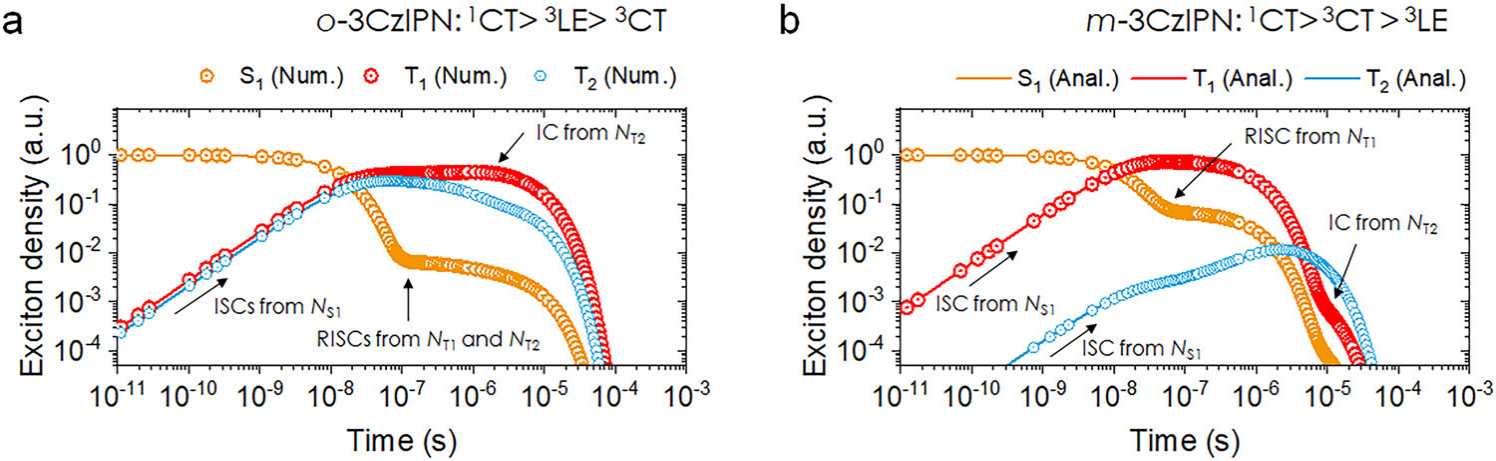
Exciton behaviors based on COMPASS model. Time-dependent population changes of excitons at T_1_, T_2,_ and S_1_ states for (**a**) *o*-3CzIPN and (**b**) *m*-3CzIPN at 300 *K*. Our model presents a kinetic description of COMPASS model, employing both numerical (symbol, Num.) and analytical (solid line, Anal.) approaches.

The time evolutions of the relative exciton density at each state further provide insights into the spin-flip process in principle. Figure 6a, b illustrate the time-dependent changes in the relative exciton density estimated at the S_1_, T_1_, and T_2_ states for *o*-3CzIPN (Fig. 6a) and *m*-3CzIPN (Fig. 6b) after optical excitation at 300 *K*, based on COMPASS model. In the case of 4CzIPN (Supplementary Fig. 23) and *o*-3CzIPN, efficient ISC occurs from the S_1_ state to both of its triplet states, T_1_ and T_2_. However, for *m*-3CzIPN, which exhibits an inverted triplet character, the ISC process occurs predominantly from S_1_ to T_1_ states. Nevertheless, this spin-flip process arises on a timescale of tens of nanoseconds, resulting in in RISC process and delayed fluorescence at a rate comparable to that of *o*-3CzIPN.

Based on COMPASS model at 77 *K* (Supplementary Figs. 24a, b), the exciton density of T_2_ state at 77 *K* becomes drastically suppressed in 4CzIPN and *o*-3CzIPN cases due to inactivation of ISC between S_1_ and T_2_ states (see also Supplementary Fig. 19). Note that the increment of exciton density for the T_2_ state relies on the inter-convertible processes via RIC at 77 *K*. In the case of *m*-3CzIPN, however, the change in exciton density at the T_2_ state at 77 *K* (Supplementary Figs. 24c) follows a response similar to that of 300 *K* (Fig. 6b) until the time range of 10^−7 ^s, although a slight deviation occurrs thereafter because the contribution from T_1_ populated via multicycles involving (T_1_$\rightarrow$S_1_$\rightarrow$T_2_) becomes minimal at 77 *K* due to the reduced RISC.

### COMPASS model and its implication on devices

**Fig. 7:**
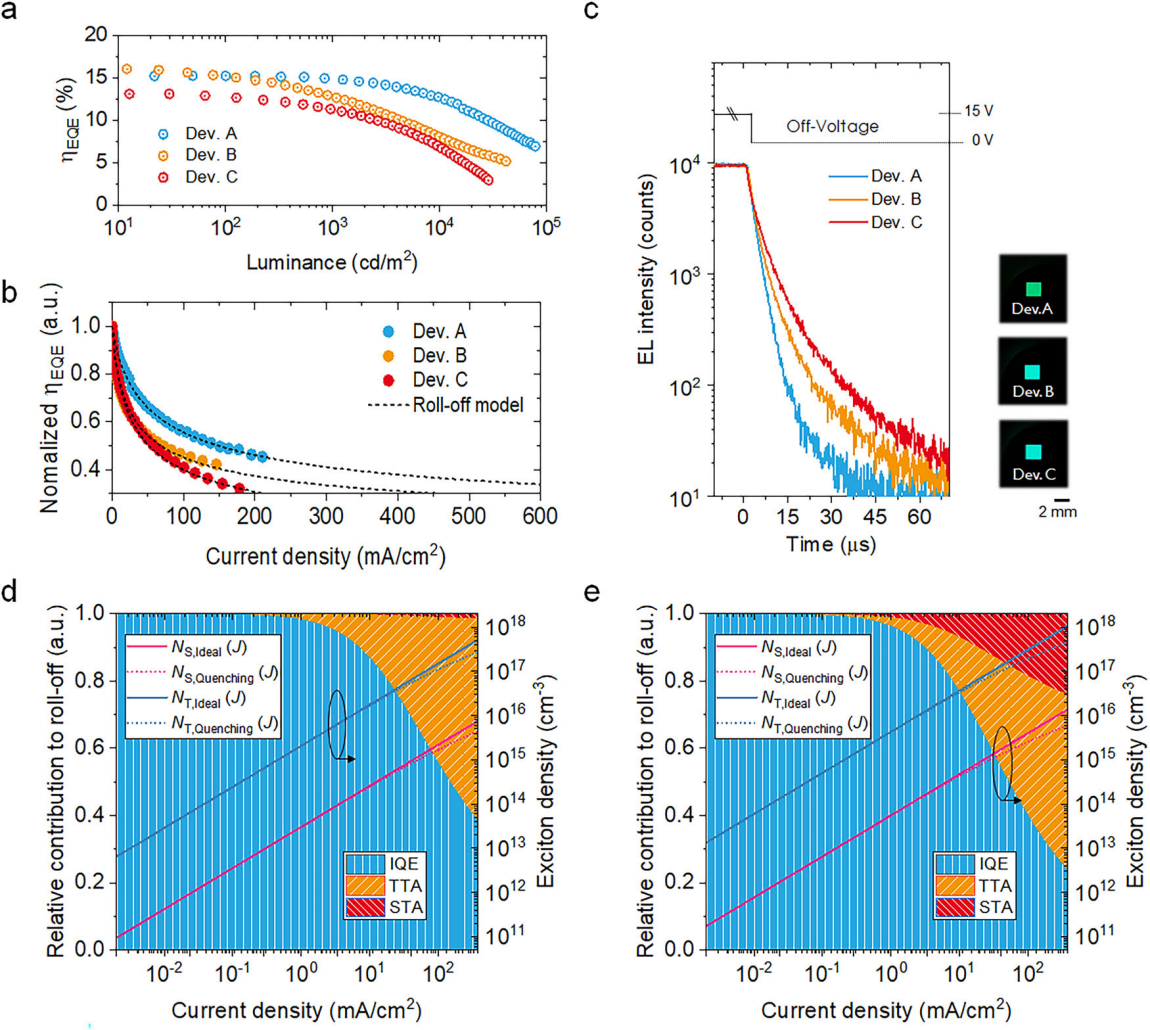
Electroluminescent (EL) characterization of IPN-derivative. **a** *η*_EQE_-*L* and **b** the normalized *η*_EQE_ vs. *J* curves, considering the bi-excitonic roll-off model (dotted line), for the tested OLED samples. **c** Transient-EL response after the square pulse excitation. (Inset image: photographs for a series of operational devices). Simulated relative contribution of TTA (orange) and STA (red) based quenching processes to efficiency roll-off as a function of *J* in the effective TADF roll-off model contracted from COMPASS model for (**d**) Dev. A (4CzIPN), and (**e**) Dev. C (*m*−3CzIPN), respectively.

There remains a question of how to relate the spin-flip process to the roll-off phenomena in the electrical system, given that for all IPN species, ${k}_{{{{{{\rm{RISC}}}}}}}^{{eff}}$ resides in the range of 10^6 ^s^−1^. To get closer to the answer, we turned our attention to IPN-based organic light-emitting diodes (OLEDs). The device architecture we designed is shown in Supplementary Fig. 25 (see also Table 1 and Supplementary Figs. 26 and 27 for a detailed summary of device characteristics). The external quantum efficiency-luminance (*η*_EQE_-*L*) curves in Fig. 7a reveal maximum EQE values of 15.2%, 16.5%, and 13.5% for Dev. A (4CzIPN), Dev. B (*o*-3CzIPN), and Dev. C (*m*-3CzIPN), respectively. We investigated the full-resolved angular EL profiles for these IPN-set shown in Supplementary Fig. 27. The obtained maximum *η*_EQE_ values are consistent with the observed photoluminescence quantum yield of the emission layer (EML) at the solid state and the horizontal molecular anisotropy (Θ_h_) summarized in Supplementary Fig. 28.

**Table. 1:** Characteristics of the tested OLED devices based on IPN-derivatives^a^

Item	V_on_^b^/V_op_^c^ (V)	CE//PE//EQE (cd/A//lm/W//%)			λ_EL_ (nm)^d^	CIE (x,y)^d^	LT_90_ (h)^e^
		Maximum	@ 1000 cd/m^2^	@10,000 cd/m^2^			
Dev. A	3.0/4.8	58.7//55.2//15.2	50.2//33.9//14.8	43.0//20.0//12.5	515	(0.295,0.589)	99
Dev. B	3.8/6.6	45.0//35.7//16.5	33.5//16.0//12.7	20.9//6.3//8.1	496	(0.206,0.459)	45
Dev. C	3.8/5.8	45.5//34.8//13.5	37.4//18.7//11.3	21.3//6.5//6.7	499	(0.211,0.474)	36

^a^ITO (100 nm)/HAT-CN (10 nm)/Tris-PCz (30 nm)/*m*CBP (5 nm)/*m*CBP:IPN-derivative (5 wt.% doped, 30 nm)/T2T (10 nm)/BPy-TP2 (40 nm)/Liq (2 nm)/Al (100 nm).

Applied voltage at luminance of ^b^1 cd/m^2^ and ^c^1000 cd/m^2^.

^d^Measured at 1000 cd/m^2^.

^e^Operational device lifetime at 90% of an initial luminance of 1000 cd/m^2^.

To provide further clarification on the role of excitonic collisions, we attempted to quantify the roll-off behavior shown in Fig. 7b from a classical perspective^36–40^. This model reflects the system contracted from COMPASS model (refer to Fig. 5a), which can be described as the three-level model we have discussed. At first glance, we have omitted the introduction of the T_2_ state into the electrical roll-off modeling, making it equivalent to considering only the effective T_1_ state (${T}_{1}^{{{{{{\rm{eff}}}}}}}$). However, this can still serve as a valid approach for investigating roll-off behavior, providing insight into the underlying spin-flip mechanism. More detailed information on the electrical roll-off model is summarized in the “Exciton dynamics” section of SI. As shown in Fig. 7b, it is evident that these curves are consistent with the bi-excitonic triplet-triplet annihilation (TTA) and singlet-triplet annihilation (STA) roll-off models^12,37^ over the entire current density (*J*) range. This observation suggests that excitonic collisions play a significant role in the roll-off behavior of the OLEDs. The rate constant for STA (${k}_{{{{{{\rm{STA}}}}}}}^{{{{{{\rm{Q}}}}}}}$) increases by one order of magnitude from Dev. A and Dev. B (1.0 × 10^−12^ cm^3^ s^−1^) to Dev. C (1.0 × 10^−11^ cm^3^ s^−1^). In contrast, the rate constant of TTA (${k}_{{{{{{\rm{TTA}}}}}}}^{{{{{{\rm{Q}}}}}}}$) is reduced in Dev. C (6.6 × 10^−13^ cm^3^ s^−1^), compared to Dev. A (1.8 × 10^−12^ cm^3^ s^−1^) and Dev. B (3.0 × 10^−12^ cm^3^ s^−1^). Note that Dev. A and Dev. B exhibit the TTA-based roll-off, while Dev. C overshadows its benefits due to the increased STA, resulting in much more severe roll-off characteristics as shown in Fig. 7b. Given that their ${k}_{{{{{{\rm{RISC}}}}}}}^{{eff}}$ are found to be within the range of 10^6 ^s^−1^ (see Supplementary Table. 5), this underscores the significance of employing COMPASS model for a comprehensive understanding of spin-flip processes in CT-type molecular systems. It can be argued that a distinction in the spin-flip pathway, particularly when it is energetically inverted within triplet manifolds, results in in distinct roll-off behaviors within the system.

The recorded Tr-EL response in Fig. 7c (see also Supplementary Fig. 29), highlights that the excitons generated by injected carriers in Dev. C require a longer time to be consumed for light emission than those of Dev A and Dev. B. The inset images in Fig. 7c correspond to the photographs of the operating devices, and it can be seen that the apparent color of each of the devices matches well with the measured EL spectra presented in Supplementary Figs. 27a–c and 29. In the steady-state modeling results presented in Fig. 7d, e and Supplementary Fig. 30, it is evident that Dev. A (Fig. 7d) and Dev. B (Supplementary Fig. 30) exhibit dominant TTA roll-off behaviors, as indicated by the orange hatched region. In contrast, Device C (Fig. 7e) follows TTA with a considerable STA quenching process, represented by the red hatched area. Despite having similar physical rate constants for *o*-3CzIPN and *m*-3CzIPN (in Supplementary Table 5), their device roll-off characteristics clearly demonstrate pronounced distinctions.

Moreover, for a given current density (*J*) in all IPN cases, the larger discrepancy between the actual (*N*_T,Quenching_) and ideal effective triplet exciton density (*N*_T,Ideal_) strongly supports the prevalence of TTA reactions at a higher current density. It is noteworthy that the concentration of triplets, represented as *N*_T_, is approximately 100 times greater than that of singlets, either *N*_S,Ideal_ or *N*_S,Quenching_ case in the steady-state. Given the highly localized nature of triplet excitons due to a small exciton diffusion length at the T_1_ state^12,40,41^, it can be thus inferred that TTA becomes predominant. This dominance is associated with the RISC process at the triplet states, leading to a reduction of delayed fluorescence as a form of exciton collision. In particular, these TTA events can even occur at low doping concentrations, such as 5.0 wt.%, owing to the effective intermolecular distance defined by center of mass (C.O.M) of 4CzIPN molecules (*d*_4CzIPN-4CzIPN_ < 18 Å). This information is revealed by the radial distribution function (RDF) based on molecular dynamic simulation, as illustrated in Supplementary Fig. 31.

The operational device stability in Supplementary Fig. 32 shows a consistent result in the order of Dev. A, Dev. B, and Dev. C as summarized in Table 1. The initial luminance (*L*_0_) for test devices was set to 1000 nits. We further conducted an investigation on the photostability of IPN sets (Supplementary Fig. 33) in comparison to the electrical case. Note that the photostability of EML comprising IPN-derivative doped in *m*CBP host film follows the order of 4CzIPN, *o*-3CzIPN, and *m*-3CzIPN. The bond dissociation energies for two key features (i.e., C$\equiv$N and C-N groups) tend to weaken as the emissive wavelength of these IPN-derivative become shorter at their respective state, as shown in Supplementary Fig. 34 and Supplementary Table 7. This supports why the molecular stability of *m*-3CzIPN, in both PL and EL cases, is weaker than the others.

As we conclude, the findings from our study shed light on the intricate spin-flip processes in a typical CT-type molecular system. The COMPASS model, encompassing a four-level structure involving the S_1_, T_2_, T_1_, and S_0_ states, has proven instrumental in unraveling the complex spin-flip routes governing exciton behaviors. Our theoretical and experimental findings demonstrate that the high-lying T state stems from a partial molecular framework in CT-type molecules, particularly in IPN-derivative. This partial electronic structure leads to a differentiation in the spin-flip pathway when it undergoes an energetic inversion within triplet manifolds. With this, we delved into the correlation between the spin-flip route and its impact on device performance in the context of the roll-off characteristics, in line with operational device stability. The COMPASS model serves as a unified and robust framework for fully comprehending exciton dynamics in CT-type molecules. We hope that this model act as a guiding compass for addressing the pressing issue discussed over the last decade.

## Methods

### Materials

In the fabrication of OLEDs, various materials were employed for this study. The hole injection layer (HIL) consisted of 1,4,5,8,9,11-hexaazatriphenylenehexacarbonitrile (HAT-CN), procured from LG Chem., Ltd. The hole transport layer (HTL) used was 9-phenyl-3,6-bis(9-phenyl-9*H*carbazol-3-yl)-9*H*-carbazole (Tris-PCz), supplied by Ooda Kasei Co., Ltd. To ensure balanced hole injection, 3,3’-di(9*H*-carbazol-9-yl)-1,1’-biphenyl (*m*CBP) was consecutively deposited as an additional HTL. *m*CBP also served as the host material for the emission layer (EML), which incorporated a series of isophthalonitrile (IPN)-derivative dopant materials; 1,2,3,5-tetrakis(carbazol-9-yl)-4,6-dicyanobenzene (4CzIPN), supplied by Chemicalsolft Co., Ltd., 4,5,6-tri(9*H*-carbazol-9-yl)isophthalonitrile (*o*-3CzIPN), and 2,4,6-tri(9*H*-carbazol-9-yl)isophthalonitrile (*m*-3CzIPN), synthesized for this work. The synthetic scheme and characterization for *o*-3CzIPN and *m*-3CzIPN, are described in our previous literature^23^. To prevent carrier overflow from EML to the electron transport layer (ETL), a hole-blocking layer (HBL) was introduced, utilizing 2,4,6-tris(biphenyl-3-yl)-1,3,5-triazine (T2T). Both the EML host and HBL materials were purchased from Nard Institute Ltd. The ETL was composed of 2,7-di(2,2’-bipyridin-5-yl)triphenylene (BPy-TP2). For the electron injection layer (EIL) and cathode, 8-Hydroxyquinolinolato-lithium (Liq) and aluminum (Al) were used, respectively. These materials were procured from Chemipro Kasei Kaisha Ltd., and Kojundo Chem., Lab., Co., Ltd.

### Optoelectronic characterization of materials

The absorption spectra of our target materials in anhydrous toluene (conc. 0.05 mM) were measured using UV/Vis/NIR spectroscopy (PerkinElmer Inc., LAMBDA 950). Room temperature photoluminescence (RTPL) and low-temperature PL (LTPL) spectra (at 77 *K*) for the target materials in both solution and solid-state cases were recorded using a PL spectrofluorometer (JASCO Inc., FP-8600). To study the transient PL (Tr-PL) behaviors for a series of TADF dopants in an Ar purged system, Tr-PL decay profiles (at *RT*) were recorded using a time-correlated single-photon counting method with a Quataurus-tau fluorescence lifetime spectrometer (Hamamatsu Photonics, C16361-02) equipped with an internal LED at 340 nm. Absolute PL quantum yields (PLQY) for the TADF materials were measured in diluted solution (conc. 0.05 mM) and in an organic thin-film deposited on quartz substrate cases (5.0 wt.% doped *m*CBP host, 50 nm thick). This was done using a Quantaurus-QY Plus UV/Vis/NIR PLQY spectrometer (Hamamatsu Photonics, C13534-11) equipped with an integrating sphere.

To investigate temperature-dependent time-resolved PL profiles in-depth, a flash-lamp pumped picosecond Nd:YAG laser (EKSPLA, PL-2250) at 355 nm was employed for the optical excitation tracer, providing 20 ps pulse duration. This ensures sufficient temporal resolution of photon decay signals (i.e., fluorescence, phosphorescence, and TADF). Photons emitted from the target material were captured by a universal streak camera (Hamamatsu Photonics, C10910-01) equipped with a spectrograph (Hamamatsu Photonics, C11119-04). In this work, we used 150 gr/mm grating with a blaze wavelength of 500 nm to achieve optimal resolution of the PL spectra. Following passage through the spectrograph, the streak tube temporally and spatially resolved the photon signals. In particular, the phosphor screens of the streak camera were rendered by a two-dimensional image, with respect to the spatial scale on the horizontal axis and time scale corresponding to the vertical axis. This process was facilitated by a high-sensitive sCMOS camera (Hamamatsu Photonics, C11440-22C). The timing of the streak sweep, micro-channel plate (MCP) gating, and the sCMOS camera operation were controlled using external trigger signals generated by a digital delay generator (Stanford Research Systems, DG645). Measurement condition were maintained at a pressure of 1.5×10^-2 ^Torr through the use of a vacuum turbo pump (Leybold Turbovac, 90i). The sample chamber, containing an organic thin-film deposited on a silicon wafer, was temperature-controlled. Starting at 50 *K*, the temperature was increased by 15 *K* intervals until reaching 300 *K*. This was done using a cryogenic temperature controller (Cryo. Con., 22 C) coupled with a helium compressor (Sumitomo, CNA-11), and cryocooler (Sumitomo, RDK-101D).

### Device characterization and measurements

Glass substrates, 25 mm × 25 mm × 0.7T, were prepared for a series of OLED sets, featuring a pre-patterned ITO electrode of 100 nm thickness. The substrates underwent a thorough cleaning process, involving sonication in deionized water, acetone, and isopropanol. Subsequently, they were irradiated in a UV-Ozone chamber to eliminate any remaining organic impurities. The organic deposition process was consecutively performed by the thermal vacuum evaporation technique, maintaining a pressure of approximately 3.7 × 10^−7 ^Torr and a deposition rate of 0.5 Å s^−1^. Liq and Al were deposited at the rates of 0.1 Å s^−1^ and 1 Å s^−1^, respectively. Following OLED fabrication, glass lids were used for the encapsulation with epoxy resin in a dry nitrogen-filled glove box with, ensuring oxygen and water content remained below 0.1 ppm. During the encapsulation procedure, we included commercially available calcium oxide desiccant (Dynic Co.) in each OLED device.

For the evaluation of current density-voltage-luminance (*J*-*V*-*L*) characteristics of OLEDs, a calibrated spectroradiometer (Konica Minolta, CS-2000) was employed in conjunction with a programmable source meter (Keithley Instruments Inc., Keithley 2400). The voltage sweep ranged from −2 V to 12 V in steps of 0.2 V. Angle-resolved EL intensity characteristics were obtained using a light distribution measurement system (Hamamatsu Photonics, C9920-11) equipped with a PMA-12 photonic multi-channel analyzer (Hamamatsu Photonics, C10027-01). Herein, we used the electrical source meter (Keithley Instruments Inc., Keithley 2601B) for the electrical driving of OLEDs at the constant current density (for a luminance of 1000 cd m^−2^). With the full angular properties (collected from −90° to +90° in steps of 5°), we corrected the values of external quantum efficiency and the power efficiency for OLED samples without Lambertian simplification.

To investigate the operational device stability of OLED samples at *RT*, device lifetimes were monitored using an OLED lifetime tester (SYSTEM ENGINEERS’ Co., LTD., EAS-26B) equipped with a spectroradiometer (TOPCON, SR-3-AR) for each individual OLED sample. The initial luminance was set to 1000 cd m^−2^ under the constant current density driving. Transient EL decay profiles of the OLED samples were collected using a streak camera (Hamamatsu Photonics, C4334) coupled with a pulse generator (Agilent, 8114A) and applying an electrical excitation with a pulse width of 20 μs and a repetition rate of 200 Hz at a constant voltage.

### Angle dependency of *p*-polarized PL profile

To investigate the molecular anisotropy (Θ) of dopants in EML, we used a molecular orientation characteristic measurement system (Hamamatsu Photonics, C13472-01). Thin films with a target thickness of 50 nm were thermally deposited on a quartz substrate and positioned at the center of a rotating stage equipped with a cylindrical lens. A certified refractive index (*n*) matching gel (CARGILLE LAB., Optical gel Code 081160, *n* = 1.517) was used between the quartz substrate and half-cylinder. Using a 350 nm LED light source unit, the spot point on the target thin film was excited, and the *p*-polarized response from the emissive species was collected by varying the emission angle (0–90°, in steps of 2°). We then determined the value of Θ by fitting the measured *p*-polarized PL profile, which is the normalized to 0°, using the classical dipole oscillation model known as C.P.S^42^. The optical simulation assumed that the emitting dipole is located at the center of the emissive layer for all cases.

### Computational details

#### Molecular electronic-structure calculation

We investigated the electronic structures of our target molecules, namely 4CzIPN, *o*-3CzIPN, and *m*-3CzIPN. Initially, we optimized the ground-state (S_0_) for each molecule using the Becke, 3-parameter, Lee-Yang-Parr (B3LYP) hybrid functional^43,44^ with a valence double-ζ (DZ) polarized basis set (6–31 G**) for all atoms^45^. We considered 6-*d* orbitals in each individual *d*-shell for a valence DZ basis set. To ensure a good description of spin-dependent excited charge-transfer (CT) characters, we employed the long-range corrected exchange functionals, Perdew-Burke-Ernzerhof exchange functional (LC-*w*PBE), along with the 6–31 G** basis set for all atoms^46^. Subsequently, we determined the optimal value of the range separation parameter (w*) by referencing the optimized ground-state geometry at the DFT level of B3LYP/6–31 G**.

This was achieved through the concept of Koopmans’ theorem, which is applicable to the (generalized) Kohn-Sham calculation^47,48^. According to Koopmans’ theorem, the value of the HOMO can be equated to the negative vertical ionization potential, neglecting orbital relaxation in the system. Similarly, the electron affinity at the *N* (*N* + 1) electron system can be considered^12,49,50^, expressing it as follows: ${J}^{2}(w)={\sum }_{i=0}^{1}{\left|{\varepsilon }_{{{{{{\rm{HOMO}}}}}}}^{w}\left(N+i\right)+{E}^{w}\left(N-1+i\right)-{E}^{w}\left(N+i\right)\right|}^{2}$. With this, we iteratively tuned the ‘gap’ for the optimal w* (Bohr^−1^) until the value of *J*^2^(*w*) became <10^−5^ eV^2^ for all cases.

Following the optimization of the ground-state geometry, the electronic vertical absorption energy (*E*_VA_) was investigated by employing tuned-LC-*w*PBE/6–31 G** at the time-dependent DFT (TD-DFT) level within the Tamm-Dancoff approximation (TDA)^51^. The first five singlet and first five triplet excited states were taken into consideration, respectively. Subsequently, the molecular geometries of the S_1_, T_1,_ and T_2_ states were optimized using tuned-LC-*w*PBE/6–31 G** at the TD-DFT level within TDA. Finally, natural transition orbital (NTO) analyses were performed to examine the nature of excited states for the target molecules^52^.

#### Spin-orbit coupling matrix element

Spin-orbit coupling (SOC) was treated as a perturbation based on the pseudo-relativistic orbitals after the self-consistent field (SCF) and TD-DFT calculation^8^. This methodology is known as pseudo-SOC-TDDFT method, and it is particularly applicable to the molecular system composed of light atoms such as C, N, and H atoms. For this purpose, we employed pseudo-SOC-TDDFT method to calculate the value of SOC matrix element using LC-w*PBE/double-ζ polarized scale of zero-order regular approximation (ZORA) basis set^53–55^ for all atoms (DYALL-2ZCVP_ZORA-J-PT-GEN) at the level of TD-DFT within TDA^56^.

#### Molecular dynamic simulation

Physically accessible disordered systems of well-equilibrated amorphous EML were prepared using the OPLS4 force-field (FF) for molecular dynamics (MD) simulation^57^. The simulation box was constructed by populating it with a uniform probability of dihedral distribution. Molecules with rotatable bonds, such as the *m*CBP host and IPN-derivatives, were re-built using a self-avoiding random walk algorithm implemented in Desmond (developed by D. E. Shaw Research)^58^. The initial placement of molecules in the cubic grid utilized a van der Waals (Vdw) scale factor of 0.5. The final clash Vdw scale factor and cell densities were confirmed at 0.5 and 0.5 g/cm^3^, respectively. For the cells with a 5.0 wt.% doped EML composition, the periodic box with a constant particle number (*N*) of 2048 molecules was used for the 4CzIPN case, and those with 1024 molecules were used for *o*-3CzIPN and *m*-3CzIPN, respectively. An MD simulation was performed using NPT (the isothermal-isobaric ensemble) class for 100 ns, employing a Nosé-Hoover chain thermostat at 300 *K* and a Martyna-Tobias-Klein barostat at 1.013 bar with isotropic pressure coupling. This MD simulation proceeded after a relaxation protocol for constructed disordered model, which included 20 ps NVT (canonical ensemble) class Brownian minimization at 10 *K*, followed by 20 ps NPT Brownian minimization at 100 *K*, and finally, 100 ps NPT MD stage at 100 *K*.

After completing the MD simulation, the convergence of simulation was confirmed, and the trajectories (i.e., a bunch of snapshots) collected from the last 20 ns were used for the analyses in this work. The predicted film densities for those systems were 1.14 g/cm^3^, with a standard deviation for density of 0.001 in all cases. All computational details in this work were carried out using Gaussian 16W^59^, Maestro materials science suite (Ver. 2022-3), the quantum chemical and DFT package Jaguar (Ver. 11.7) developed by Schrödinger Inc^60^., ORCA (Ver. 5.0.3)^61^, and a multifunctional wave function software, Multiwfn (Ver. 3.8)^62^.

### Supplementary information

Supplementary Information

Peer Review File

Supplementary Movie 1: Molecular dynamics simulation trajectory for a 5 wt. % 4CzIPN-doped mCBP host film

### Source data

Source Data

## Data Availability

The authors declare that the data supporting the findings of this study are available within the article and its Supplementary Information files. Numerical values of data shown as graphs are available from the corresponding authors. Source data have been deposited in the FigShare digital data repository under accession code^63^; 10.6084/m9.figshare.c.7058540. Source data are provided with this paper.
