# Supplementary material for: Understanding of complex spin up-conversion processes in charge-transfer-type organic molecules
Source: Nat Commun. 2024 Mar 13;15:2267. doi: 10.1038/s41467-024-46406-5 (PMC10937997; doi:10.1038/s41467-024-46406-5)
Supplement: Supplementary file 1 — Supplementary Information [file 41467_2024_46406_MOESM1_ESM.pdf]

Supplementary Information

Understanding of Complex Spin Up-conversion Processes in Charge-Transfer-Type Organic Molecules

Hyung Suk Kim[1,2]†, Sang Hoon Lee[2]†, Seunghyup Yoo[3]*, Chihaya Adachi[1,2,4]*

Affiliations:

[1]Center for Organic Photonics and Electronics Research (OPERA), Kyushu University, 744 Motooka, Nishi, Fukuoka, 819-0395, Japan

[2]Department of Applied Chemistry, Kyushu University, 744 Motooka, Nishi, Fukuoka, 819-0395, Japan

[3]School of Electrical Engineering, Korea Advanced Institute of Science and Technology (KAIST), Daejeon 34141, Republic of Korea

[4]International Institute for Carbon Neutral Energy Research (I^{2}CNER), Kyushu University, 744 Motooka, Nishi, Fukuoka, 819-0395, Japan

†These authors contributed equally to this work.

*Corresponding author.

Corresponding author: syoo.ee@kaist.edu, adachi@cstf.kyushu-u.ac.jp

The PDF file includes:

A. Exciton Dynamics
B. Abbreviation
C. Fundamental Photophysical Properties
D. Computational Results
E. Rate Relation Based on Exciton Dynamics
F. Device Characterization
G. Operational PL/EL Stability Test

Supplementary Figs. 1 to 34
Supplementary Tables 1 to 7
References

A. Exciton Dynamics

I. Temperature dependency of the delayed component (k_d) behavior. Within the framework of the optical three-level model (i.e., S_1, T_1, and S_0 state), the exciton model based on time (t)-dependent population rate equations can be expressed as follows[1-2];

$$\frac{dS_1(t)}{dt} = -(k_r^s + k_{nr}^s + k_{ISC})S_1(t) + k_{RISC}T_1(t) + G$$

Eq. (S1)

$$\frac{dT_1(t)}{dt} = -(k_{nr}^T + k_r^T + k_{RISC})T_1(t) + k_{ISC}S_1(t)$$

Eq. (S2)

The symbol G in equation (S1) represents the singlet exciton generation rate (cm^{-3} s^{-1}) resulting from the optical pumping. Using **Eqs.** (S1) and (S2), we can come by the analytical solutions for these rate equations. As a result, the rate constant of the delayed component (k_d) for describing the PL decay profile is thus given by;

$$k_d^{S_1 \leftrightarrow T_1}(T) = \frac{(A' + B') - \sqrt{(A' + B')^2 - 4[A'B' - k_{ISC}k_{RISC}(T)]}}{2}$$

Eq. (S3)

Wherein the components A' and B' correspond to $k_r^s + k_{nr}^s + k_{ISC}$ and $k_r^T + k_{nr}^T + k_{RISC}(T)$, respectively. It is noted that temperature (T) dependent k_{RISC} can be defined by the following relation (i.e., Arrhenius equation)[1-2];

$$k_{RISC}(T) = k_{ISC}e^{-\frac{\Delta E_{S_1-T_1}}{k_B T}}$$

Eq. (S4)

Here, k_B is the Boltzmann constant, and $\Delta E_{S_1-T_1}$ represents the energy difference between S_1 and T_1 state. Therefore, by incorporating the temperature-dependent component, given by **Eq.** (S4), into **Eq.** (S3), we can characterize the temperature-dependent response of k_d (s^{-1}) response, as indicated by **Eq.** (S3).

Unfortunately, this model is limited to cover the entire system with temperature dependency, as illustrated in **Figs. 4** a-c (main text). To address this limitation, we explored two possible three-level models — *System A* (comprising T_1, T_2, and S_0 states) and *System B* (consisting of S_1, T_2, and S_0 states) — to elucidate exciton behaviors over a wide temperature range. It is important to note that, for each system, excluded states are explicitly disregarded in the respective model. For instance, in the case of *System A*, the S_1 state does not interact with the T_1, T_2, and S_0 states. Similarly, for System B, the T_1 state is presumed to have no rate constants associated with interactions involving the S_1, T_2, and S_0 states.

It is worth noting that even when the particle undergoes the same changes with different kinetic parameters based on the time-dependent population of rate equations, the resulting mathematical solutions, such as **Eq.** (S3), remain consistent.

We thereby derive **Eq.** (S5) for k_d (s^{-1}) for $T_1 \leftrightarrow T_2$ interaction (*System A)*,

$$k_d^{T_1 \leftrightarrow T_2}(T) = \frac{(A'' + B'') - \sqrt{(A'' + B'')^2 - 4[A''B'' - k_{IC}^{T_2 \rightarrow T_1} k_{RIC}^{T_1 \rightarrow T_2}(T)]}}{2}$$

Eq. (S5)

The components A'' and B'' correspond to $k_r^{T_2 \rightarrow S_0} + k_{IC}^{T_2 \rightarrow T_1}$ and $k_{RIC}^{T_1 \rightarrow T_2}(T) + k_r^{T_1 \rightarrow S_0} + k_{nr}^{T_1 \rightarrow S_0}$, respectively. Similarly, we employed a Boltzmann distribution to describe the population of excited-state (i.e., T_1 and T_2 state), given by;

$$k_{RIC}^{T_1 \rightarrow T_2}(T) = k_{IC}^{T_2 \rightarrow T_1} e^{-\frac{\Delta E_{T_2 - T_1}}{k_B T}}$$

Eq. (S6)

By employing the same approach, we can obtain an equation for k_d (s^{-1}) for $S_1 \leftrightarrow T_2$ interaction (*System B*)*,* as follows:

$$k_d^{S_1 \leftrightarrow T_2}(T) = \frac{(A''' + B''') - \sqrt{(A''' + B''')^2 - 4[A'''B''' - k_{ISC}^{S_1 \rightarrow T_2} k_{RISC}^{T_2 \rightarrow S_1}(T)]}}{2}$$

Eq. (S7)

where A''' and B''' are designed as $k_r^{S_1 \rightarrow S_0} + k_{ISC}^{S_1 \rightarrow T_2} + k_{nr}^{S_1 \rightarrow S_0}$ and $k_{RISC}^{T_2 \rightarrow S_1}(T) + k_r^{T_2 \rightarrow T_1} + k_{nr}^{T_2 \rightarrow S_0}$, respectively. The temperature dependency of k_d (s^{-1}) in the system, considering a Boltzmann distribution for the population of excited-state between S_1 and T_2 state, can be expressed as;

$$k_{RISC}^{T_2 \rightarrow S_1}(T) = k_{ISC}^{S_1 \rightarrow T_2} e^{-\frac{\Delta E_{S_1 - T_2}}{k_B T}}$$

Eq. (S8)

II. Derivation of COMPASS model (optical pumping). As previously mentioned, we observed that the three-level model was not enough to explain the experimental TADF PL decay responses at all temperatures in this work. Specifically, it is noted that a sum of bi-exponential decay, derived from the differential equations [i.e., **Eqs.** (S1) and (S2)], could only capture a portion of the exciton behaviors in the TADF molecular system within the particular temperature range (refer to **Figs. 4** a-c in the main text).

In response to this limitation, we introduced COMPASS model (see **Fig. 2** depicted in the main text), which includes the four-level model (i.e., S_1, T_2, T_1, and S_0 state). This model is further developed one based on a prior model by Kobayashi et al.[3]. Subsequently, we then describe the time evolution of exciton densities at each excited-state (i.e., S_1, T_2, and T_1 state) by employing a series of differential equations, as presented below;

$$\frac{d\mathrm{S}_1(t)}{dt} = -\left(k_{\mathrm{r}}^{\mathrm{S}_1\rightarrow\mathrm{S}_0} + k_{\mathrm{nr}}^{\mathrm{S}_1\rightarrow\mathrm{S}_0} + k_{\mathrm{ISC}}^{\mathrm{S}_1\rightarrow\mathrm{T}_2} + k_{\mathrm{ISC}}^{\mathrm{S}_1\rightarrow\mathrm{T}_1}\right)\mathrm{S}_1(t) + k_{\mathrm{RISC}}^{\mathrm{T}_2\rightarrow\mathrm{S}_1}\mathrm{T}_2(t) + k_{\mathrm{RISC}}^{\mathrm{T}_1\rightarrow\mathrm{S}_1}\mathrm{T}_1(t) + G$$

Eq. (S9)

$$\frac{d\mathrm{T}_2(t)}{dt} = k_{\mathrm{ISC}}^{\mathrm{S}_1\rightarrow\mathrm{T}_2}\mathrm{S}_1(t) - \left(k_{\mathrm{r}}^{\mathrm{T}_2\rightarrow\mathrm{S}_0} + k_{\mathrm{IC}}^{\mathrm{T}_2\rightarrow\mathrm{T}_1} + k_{\mathrm{RISC}}^{\mathrm{T}_2\rightarrow\mathrm{S}_1}\right)\mathrm{T}_2(t) + k_{\mathrm{RIC}}^{\mathrm{T}_1\rightarrow\mathrm{T}_2}\mathrm{T}_1(t)$$

Eq. (S10)

$$\frac{d\mathrm{T}_1(t)}{dt} = k_{\mathrm{ISC}}^{\mathrm{S}_1\rightarrow\mathrm{T}_1}\mathrm{S}_1(t) + k_{\mathrm{IC}}^{\mathrm{T}_2\rightarrow\mathrm{T}_1}\mathrm{T}_2(t) - \left(k_{\mathrm{r}}^{\mathrm{T}_1\rightarrow\mathrm{S}_0} + k_{\mathrm{nr}}^{\mathrm{T}_1\rightarrow\mathrm{S}_0} + k_{\mathrm{RISC}}^{\mathrm{T}_1\rightarrow\mathrm{S}_1} + k_{\mathrm{RIC}}^{\mathrm{T}_1\rightarrow\mathrm{T}_2}\right)\mathrm{T}_1(t)$$

Eq. (S11)

For the simplification of **Eqs.** (S9) to (S11), let a sum of rate-constant as *A*, *B*, and *C* as follows;

$$A = k_{\mathrm{r}}^{\mathrm{S}_1\rightarrow\mathrm{S}_0} + k_{\mathrm{nr}}^{\mathrm{S}_1\rightarrow\mathrm{S}_0} + k_{\mathrm{ISC}}^{\mathrm{S}_1\rightarrow\mathrm{T}_2} + k_{\mathrm{ISC}}^{\mathrm{S}_1\rightarrow\mathrm{T}_1}$$

Eq. (S12)

$$B = k_{\mathrm{r}}^{\mathrm{T}_2\rightarrow\mathrm{S}_0} + k_{\mathrm{IC}}^{\mathrm{T}_2\rightarrow\mathrm{T}_1} + k_{\mathrm{RISC}}^{\mathrm{T}_2\rightarrow\mathrm{S}_1}$$

Eq. (S13)

$$C = k_{\mathrm{r}}^{\mathrm{T}_1\rightarrow\mathrm{S}_0} + k_{\mathrm{nr}}^{\mathrm{T}_1\rightarrow\mathrm{S}_0} + k_{\mathrm{RISC}}^{\mathrm{T}_1\rightarrow\mathrm{S}_1} + k_{\mathrm{RIC}}^{\mathrm{T}_1\rightarrow\mathrm{T}_2}$$

Eq. (S14)

Herein, *A*, *B*, and *C* correspond to the sum of total exciton consumption routes (s^{-1}) at S_1, T_2, and T_1 states, respectively. Additionally, we considered the temperature dependence of rate constants using Arrhenius equation. This incorporates the activation energy of ISC ($\Delta E_{\mathrm{A}}^{\mathrm{ISC}}$) as well as energy differences (ΔE) between excited states, and can be written as follows;

$$k_{\mathrm{ISC}}^{\mathrm{S}_1\rightarrow\mathrm{T}_2}(T) = k_{\mathrm{ISC}}^{\mathrm{S}_1\rightarrow\mathrm{T}_2} e^{-\frac{\Delta E_{\mathrm{A}}^{\mathrm{ISC}}}{k_{\mathrm{B}}(T-T_{\mathrm{Onset}})}}$$

Eq. (S15)

Here, the energy barrier required for ISC from S_1 to T_2 state, along with the point at which ISC initiates at a specific temperature, T_{Onset}, is defined.

$$k_{\mathrm{RISC}}^{\mathrm{T}_2\rightarrow\mathrm{S}_1}(T) = k_{\mathrm{ISC}}^{\mathrm{S}_1\rightarrow\mathrm{T}_2} e^{-\frac{\Delta \mathrm{E}_{\mathrm{S}_1-\mathrm{T}_2}}{k_{\mathrm{B}}T}}$$

Eq. (S16)

$$k_{\mathrm{RISC}}^{\mathrm{T}_1\rightarrow\mathrm{S}_1}(T) = k_{\mathrm{ISC}}^{\mathrm{S}_1\rightarrow\mathrm{T}_1} e^{-\frac{\Delta \mathrm{E}_{\mathrm{S}_1-\mathrm{T}_1}}{k_{\mathrm{B}}T}}$$

Eq. (S17)

$$k_{\mathrm{RIC}}^{\mathrm{T}_1\rightarrow\mathrm{T}_2}(T) = k_{\mathrm{IC}}^{\mathrm{T}_2\rightarrow\mathrm{T}_1} e^{-\frac{\Delta \mathrm{E}_{\mathrm{T}_2-\mathrm{T}_1}}{k_{\mathrm{B}}T}}$$

Eq. (S18)

Then, we can take Laplace transform of **Eqs.** (S9) to (S11) for the derivation of analytical solutions of COMPASS model, which is given by;

$$\mathcal{L}\left\{\frac{d\mathrm{S}_1(t)}{dt}\right\}(s) = -AX(s) + k_{\mathrm{RISC}}^{\mathrm{T}_2\rightarrow\mathrm{S}_1}Y(s) + k_{\mathrm{RISC}}^{\mathrm{T}_1\rightarrow\mathrm{S}_1}Z(s)$$

Eq. (S19)

$$\mathcal{L}\left\{\frac{d\mathrm{T}_2(t)}{dt}\right\}(s) = k_{\mathrm{ISC}}^{\mathrm{S}_1\rightarrow\mathrm{T}_2}X(s) - BY(s) + k_{\mathrm{RIC}}^{\mathrm{T}_1\rightarrow\mathrm{T}_2}Z(s)$$

Eq. (S20)

$$\mathcal{L}\left\{\frac{d\mathrm{T}_1(t)}{dt}\right\}(s) = k_{\mathrm{ISC}}^{\mathrm{S}_1\rightarrow\mathrm{T}_1}X(s) + k_{\mathrm{IC}}^{\mathrm{T}_2\rightarrow\mathrm{T}_1}Y(s) - CZ(s)$$

Eq. (S21)

By the definition of Laplace transform, hereby, $\mathcal{L}\{\mathrm{S}_1(t)\}(s) \equiv X(s)$, $\mathcal{L}\{\mathrm{T}_2(t)\}(s) \equiv Y(s)$, and $\mathcal{L}\{\mathrm{T}_1(t)\}(s) \equiv Z(s)$. To solve COMPASS model, we take the boundary conditions for the optical pumping case; $\mathrm{S}_1(0) = 1$, $\mathrm{T}_2(0) = 0$, and $\mathrm{T}_1(0) = 0$, corresponding to the generated singlet excitons at S_1 state from the photon absorption (i.e., *G* is normalized to the value of unity). With this, we can formulate **Eqs.** (S19) to (S21) as a system of linear equations in the *s*-domain (i.e., Laplace space).

$$\begin{bmatrix} s+A & -k_{\mathrm{RISC}}^{\mathrm{T}_2\rightarrow\mathrm{S}_1} & -k_{\mathrm{RISC}}^{\mathrm{T}_1\rightarrow\mathrm{S}_1} \\ -k_{\mathrm{ISC}}^{\mathrm{S}_1\rightarrow\mathrm{T}_2} & s+B & -k_{\mathrm{RIC}}^{\mathrm{T}_1\rightarrow\mathrm{T}_2} \\ -k_{\mathrm{ISC}}^{\mathrm{S}_1\rightarrow\mathrm{T}_1} & -k_{\mathrm{IC}}^{\mathrm{T}_2\rightarrow\mathrm{T}_1} & s+C \end{bmatrix} \begin{bmatrix} X(s) \\ Y(s) \\ Z(s) \end{bmatrix} = \begin{bmatrix} 1 \\ 0 \\ 0 \end{bmatrix}$$

Eq. (S22)

By solving **Eq.** (S22), we then come by;

$$X(s) = \frac{(s+B)(s+C) - k_{\mathrm{RIC}}^{\mathrm{T_1 \to T_2}} k_{\mathrm{IC}}^{\mathrm{T_2 \to T_1}}}{(s+k_1)(s+k_2)(s+k_3)}$$

Eq. (S23)

$$Y(s) = \frac{(s+B) k_{\mathrm{ISC}}^{\mathrm{S_1 \to T_2}} + k_{\mathrm{RIC}}^{\mathrm{T_1 \to T_2}} k_{\mathrm{ISC}}^{\mathrm{S_1 \to T_1}}}{(s+k_1)(s+k_2)(s+k_3)}$$

Eq. (S24)

$$Z(s) = \frac{(s+C) k_{\mathrm{ISC}}^{\mathrm{S_1 \to T_1}} + k_{\mathrm{IC}}^{\mathrm{T_2 \to T_1}} k_{\mathrm{ISC}}^{\mathrm{S_1 \to T_2}}}{(s+k_1)(s+k_2)(s+k_3)}$$

Eq. (S25)

Herein, $(s+k_1)(s+k_2)(s+k_3) = s^3 + \alpha s^2 + \beta s + \gamma$ where -k_1, -k_2, and -k_3 are the solution of the cubic equation in *s*-domain. Using Viète's theorem, the following relation can be derived as follows [refer to **Eqs.** (S12) to (S14) for the definition of *A*, *B*, and *C*];

$$\alpha = A + B + C$$

Eq. (S26)

$$\beta = AB + AC + BC - k_{\mathrm{RIC}}^{\mathrm{T_1 \to T_2}} k_{\mathrm{IC}}^{\mathrm{T_2 \to T_1}} - k_{\mathrm{RISC}}^{\mathrm{T_1 \to S_1}} k_{\mathrm{ISC}}^{\mathrm{S_1 \to T_1}} - k_{\mathrm{RISC}}^{\mathrm{T_2 \to S_1}} k_{\mathrm{ISC}}^{\mathrm{S_1 \to T_2}}$$

Eq. (S27)

$$\gamma = ABC - A k_{\mathrm{RIC}}^{\mathrm{T_1 \to T_2}} k_{\mathrm{IC}}^{\mathrm{T_2 \to T_1}} - B k_{\mathrm{RISC}}^{\mathrm{T_2 \to S_1}} k_{\mathrm{ISC}}^{\mathrm{S_1 \to T_2}} - C k_{\mathrm{RISC}}^{\mathrm{T_1 \to S_1}} k_{\mathrm{ISC}}^{\mathrm{S_1 \to T_1}} - k_{\mathrm{IC}}^{\mathrm{T_2 \to T_1}} k_{\mathrm{ISC}}^{\mathrm{S_1 \to T_2}} k_{\mathrm{RISC}}^{\mathrm{T_1 \to S_1}} - k_{\mathrm{ISC}}^{\mathrm{S_1 \to T_1}} k_{\mathrm{RIC}}^{\mathrm{T_1 \to T_2}} k_{\mathrm{RISC}}^{\mathrm{T_2 \to S_1}}$$

Eq. (S28)

For the inverse Laplace transform of **Eqs.** (S23) to (S25), we can use the partial fraction decomposition with *Heaviside* cover-up method, and the solutions of a series of rate equations in the time (*t*) domain are derived;

$$\mathrm{S_1}(t) = \frac{(B-k_1)(C-k_1) - k_{\mathrm{RIC}}^{\mathrm{T_1 \to T_2}} k_{\mathrm{IC}}^{\mathrm{T_2 \to T_1}}}{(k_2-k_1)(k_3-k_1)} e^{-k_1 t} + \frac{(B-k_2)(C-k_2) - k_{\mathrm{RIC}}^{\mathrm{T_1 \to T_2}} k_{\mathrm{IC}}^{\mathrm{T_2 \to T_1}}}{(k_1-k_2)(k_3-k_2)} e^{-k_2 t} + \frac{(B-k_3)(C-k_3) - k_{\mathrm{RIC}}^{\mathrm{T_1 \to T_2}} k_{\mathrm{IC}}^{\mathrm{T_2 \to T_1}}}{(k_1-k_3)(k_2-k_3)} e^{-k_3 t}$$

Eq. (S29)

$$\mathrm{T_2}(t) = \frac{(B-k_1) k_{\mathrm{ISC}}^{\mathrm{S_1 \to T_2}} + k_{\mathrm{RIC}}^{\mathrm{T_1 \to T_2}} k_{\mathrm{ISC}}^{\mathrm{S_1 \to T_1}}}{(k_2-k_1)(k_3-k_1)} e^{-k_1 t} + \frac{(B-k_2) k_{\mathrm{ISC}}^{\mathrm{S_1 \to T_2}} + k_{\mathrm{RIC}}^{\mathrm{T_1 \to T_2}} k_{\mathrm{ISC}}^{\mathrm{S_1 \to T_1}}}{(k_1-k_2)(k_3-k_2)} e^{-k_2 t} + \frac{(B-k_3) k_{\mathrm{ISC}}^{\mathrm{S_1 \to T_2}} + k_{\mathrm{RIC}}^{\mathrm{T_1 \to T_2}} k_{\mathrm{ISC}}^{\mathrm{S_1 \to T_1}}}{(k_1-k_3)(k_2-k_3)} e^{-k_3 t}$$

Eq. (S30)

$$\mathrm{T}_1(t) = \frac{(C-k_1)k_{\mathrm{ISC}}^{\mathrm{S}_1\to\mathrm{T}_1} + k_{\mathrm{IC}}^{\mathrm{T}_2\to\mathrm{T}_1}k_{\mathrm{ISC}}^{\mathrm{S}_1\to\mathrm{T}_2}}{(k_2-k_1)(k_3-k_1)}e^{-k_1 t} + \frac{(C-k_2)k_{\mathrm{ISC}}^{\mathrm{S}_1\to\mathrm{T}_1} + k_{\mathrm{IC}}^{\mathrm{T}_2\to\mathrm{T}_1}k_{\mathrm{ISC}}^{\mathrm{S}_1\to\mathrm{T}_2}}{(k_1-k_2)(k_3-k_2)}e^{-k_2 t} + \frac{(C-k_3)k_{\mathrm{ISC}}^{\mathrm{S}_1\to\mathrm{T}_1} + k_{\mathrm{IC}}^{\mathrm{T}_2\to\mathrm{T}_1}k_{\mathrm{ISC}}^{\mathrm{S}_1\to\mathrm{T}_2}}{(k_1-k_3)(k_2-k_3)}e^{-k_3 t}$$

Eq. (S31)

Note that the constants, k_1, k_2, and k_3 used in this work are real numbers, all greater than 0 (s^{-1}). In this study, we have provided all physical constants for COMPASS model. However, we have assumed that the non-radiative exciton decays, particularly for $k_{\mathrm{nr}}^{\mathrm{S}_1\to\mathrm{S}_0}$ and $k_{\mathrm{nr}}^{\mathrm{T}_1\to\mathrm{S}_0}$, are both set to 0 s^{-1}. We observed that the thermal dissipation process associated with $k_{\mathrm{nr}}^{\mathrm{S}_1\to\mathrm{S}_0}$ in the S_1 state is insufficient to change for the system at all temperatures (i.e., 50 *K* to 300 *K*). It is important to note that the rapid singlet exciton consumption occurs in the presence of fluorescence and a pair of ISC routes (refer to **Fig. 6** in the main text).

Additionally, the LTPL responses of IPN sets at low temperatures support that the necessity for the rate constants of radiative triplet exciton decay to be efficient in order to have a probability of existence, especially when compared to those of non-radiative triplet decays, such as $k_{\mathrm{IC}}^{\mathrm{T}_2\to\mathrm{T}_1}$ and $k_{\mathrm{nr}}^{\mathrm{T}_1\to\mathrm{S}_0}$. Importantly, we may speculate that the rate constant of non-radiative triplet decay ($k_{\mathrm{nr}}^{\mathrm{T}}$) based on the three-level model actually corresponds to that of radiative triplet decay (associated with a considerable ^{3}LE character to induce phosphorescence; $k_{\mathrm{r}}^{\mathrm{T}_1\to\mathrm{S}_0}$ or $k_{\mathrm{r}}^{\mathrm{T}_2\to\mathrm{S}_0}$) in COMPASS model, thereby resulting in an efficient phosphorescence. Refer to $k_{\mathrm{r}}^{\mathrm{T}_2\to\mathrm{S}_0}$ for 4CzIPN and *o*-3CzIPN, and $k_{\mathrm{r}}^{\mathrm{T}_1\to\mathrm{S}_0}$ for *m*-3CzIPN.

The definitions of terms related to the entire set of equations in this work are extensively described in **Abbreviation** section.

III. Contracted COMPASS model and its roll-off analysis (electrical excitation). To gain a better understanding of the electrical TADF system with roll-off behavior, we extended our model to include bi-excitonic annihilations, singlet-triplet annihilation (STA) and triplet-triplet annihilation (TTA), which are known contributors to the critical roll-off in TADF-based OLED devices.[4-5] We recently discovered that the origin of roll-off behavior in the 4CzIPN-doped film (*m*CBP host, 5.0 wt.% doped), under the same fabrication condition as in this work, is attributed to STA and TTA annihilation processes under the optical pumping.[5]

By employing a series of findings that we have discussed, we developed an effective (eff.) TADF roll-off model under the steady-state condition by simplifying COMPASS model (i.e., the four-level) to the three-level (as commonly used).[1-2] This enable us to assess the appropriateness of using the three-level model at room temperature by introducing the effective rate constant of ISC ($k_{\mathrm{ISC}}^{\mathrm{eff}}$) and RISC ($k_{\mathrm{RISC}}^{\mathrm{eff}}$) using **Eqs.** (6) and (7) in the main text. This means we regarded the T_1 and T_2 states as a single effective triplet state ($T_{1,\mathrm{eff}}$). In the same context, we can designate S_1 state at COMPASS model as a single effective singlet state ($S_{1,\mathrm{eff}}$). Thereby, we can come by;

$$\frac{dS_{1,\mathrm{eff}}(t)}{dt} = -\left(k_{\mathrm{r}}^{\mathrm{S_1\rightarrow S_0}} + k_{\mathrm{ISC}}^{\mathrm{eff}}\right)\mathrm{S}_{1,\mathrm{eff}}(t) + k_{\mathrm{RISC}}^{\mathrm{eff}}\mathrm{T}_{1,\mathrm{eff}}(t) - k_{\mathrm{STA}}^{Q}S_{1,\mathrm{eff}}(t)T_{1,\mathrm{eff}}(t) + \frac{1}{8}k_{\mathrm{TTA}}^{\mathrm{Q}}T_{1,\mathrm{eff}}(t)^2 + \frac{J}{4qd}$$

Eq. (S32)

$$\frac{d\mathrm{T}_{1,\mathrm{eff}}(t)}{dt} = k_{\mathrm{ISC}}^{\mathrm{eff}}\mathrm{S}_{1,\mathrm{eff}}(t) - \left(k_{\mathrm{r}}^{\mathrm{T_n\rightarrow S_0}} + k_{\mathrm{RISC}}^{\mathrm{eff}}\right)\mathrm{T}_{1,\mathrm{eff}}(t) - \frac{5}{8}k_{\mathrm{TTA}}^{Q}\mathrm{T}_{1,\mathrm{eff}}(t)^2 + \frac{3J}{4qd}$$

Eq. (S33)

Herein, the rate constant of $k_{\mathrm{STA}}^{\mathrm{Q}}$ and $k_{\mathrm{TTA}}^{\mathrm{Q}}$ ($\mathrm{cm^3\ s^{-1}}$) denote the bi-excitonic quenching process, STA and TTA processes, respectively. In this study, we adopted a singlet production ratio (α) of 0.25 according to spin-statistics. For the triplet case, the exothermic reaction from TTA occurs by a factor of half, thereby resulting in the triplet attenuation ratio of -5/8, defined as 1 – (1 – α)/2. *J* is the current density ($\mathrm{mA/cm^2}$), and *q* is the elementary charge. We took the thickness of recombination zone (*d*) is equal to that of EML (i.e., 30 nm) in this work.

Note that the rate constant of non-radiative triplet decay ($k_{\mathrm{nr}}^{\mathrm{T}}$) based on the three-level model[2] corresponds to that of efficient radiative triplet decay ($k_{\mathrm{r}}^{\mathrm{T_1\rightarrow S_0}}$ or $k_{\mathrm{r}}^{\mathrm{T_2\rightarrow S_0}}$) in this simplified COMPASS model [i.e., $k_{\mathrm{r}}^{\mathrm{T_n\rightarrow S_0}}$ in **Eq.** (S33)]. Hereby, we used $k_{\mathrm{nr}}^{\mathrm{T}}$ for $k_{\mathrm{r}}^{\mathrm{T_2\rightarrow S_0}}$ in 4CzIPN and *o*-3CzIPN cases, and

$k_{\mathrm{r}}^{\mathrm{T_1 \rightarrow S_0}}$ for *m*-3CzIPN one, respectively. All rate constants for IPN-set for this effective COMPASS model were used from **Supplementary Tables. 5-6**.

In conclusion, the forms of **Eqs.** (S32) and (S33) are similar to the model suggested by Masui et al., as referenced.[4] This model is currently acknowledged as a seminal TADF roll-off model and is widely recognized for its precision in characterizing roll-off behavior in TADF-based OLEDs.

We also note that the parameters used here are effectively applicable as rate constants, as COMPASS model can be contracted into a general three-level model, justifying their use (refer to **Supplementary Tables 4-5**).

B. Abbreviation

(I) The rate constants and efficiencies for the three-level model.

#. The rate constants for the three-level model

$k_{\mathrm{r}}^{\mathrm{S}}$: The rate constant of radiative singlet decay.

$k_{\mathrm{nr}}^{\mathrm{S}}$: The rate constant of non-radiative singlet decay.

k_{ISC}: The rate constant of intersystem crossing (ISC) from S_1 to T_1 state.

k_{RISC}: The rate constant of reverse intersystem crossing (RISC) from T_1 to S_1 state.

$k_{\mathrm{r}}^{\mathrm{T}}$: The rate constant of radiative triplet decay.

$k_{\mathrm{nr}}^{\mathrm{T}}$: The rate constant of non-radiative triplet decay.

#. The efficiencies for the three-level model

Φ_{PLQY}: The photoluminescence quantum yield (PLQY) of the system.

Φ_{PF}: The prompt fluorescence quantum yield.

Φ_{DF}: The delayed fluorescence quantum yield.

Φ_{ISC}: The efficiency of ISC.

Φ_{RISC}: The efficiency of RISC.

(II) The rate constants for COMPASS model.

$k_{\mathrm{r}}^{\mathrm{S_1 \to S_0}}$: The rate constant of radiative singlet decay.
$k_{\mathrm{nr}}^{\mathrm{S_1 \to S_0}}$: The rate constant of non-radiative singlet decay.
$k_{\mathrm{ISC}}^{\mathrm{S_1 \to T_1}}$: The rate constant of ISC from S_1 to T_1 state.
$k_{\mathrm{ISC}}^{\mathrm{S_1 \to T_2}}$: The rate constant of ISC from S_1 to T_2 state.

$k_{\mathrm{r}}^{\mathrm{T_1 \to S_0}}$: The rate constant of radiative triplet decay @ T_1 state.
$k_{\mathrm{nr}}^{\mathrm{T_1 \to S_0}}$: The rate constant of non-radiative triplet decay @ T_1 state.
$k_{\mathrm{RISC}}^{\mathrm{T_1 \to S_1}}$: The rate constant of RISC from T_1 to S_1 state.
$k_{\mathrm{RIC}}^{\mathrm{T_1 \to T_2}}$: The rate constant of reverse internal conversion (RIC) from T_1 to T_2 state.

$k_{\mathrm{r}}^{\mathrm{T_2 \to S_0}}$: The rate constant of radiative triplet decay @ T_2 state.
$k_{\mathrm{IC}}^{\mathrm{T_2 \to T_1}}$: The rate constant of internal conversion (IC) from T_2 to T_1 state.
$k_{\mathrm{RISC}}^{\mathrm{T_2 \to S_1}}$: The rate constant of RISC from T_2 to S_1 state.

k_{ISC}^{eff} : The total (effective) rate constant of ISC processes from S_1 state to T_1 and T_2 states.
k_{RISC}^{eff} : The total (effective) rate constant of RISC processes from T_1 and T_2 states to S_1 state.

$\Delta E_{\mathrm{A}}^{\mathrm{ISC}}$: The activation energy for ISC from S_1 to T_2 state.
$\Delta E_{\mathrm{S_1-T_1}}$: The energy difference (Δ) between S_1 and T_1 state.
$\Delta E_{\mathrm{S_1-T_2}}$: The energy difference between S_1 and T_2 state.
$\Delta E_{\mathrm{T_2-T_1}}$: The energy difference between T_2 and T_1 state.

C. Fundamental Photophysical Properties

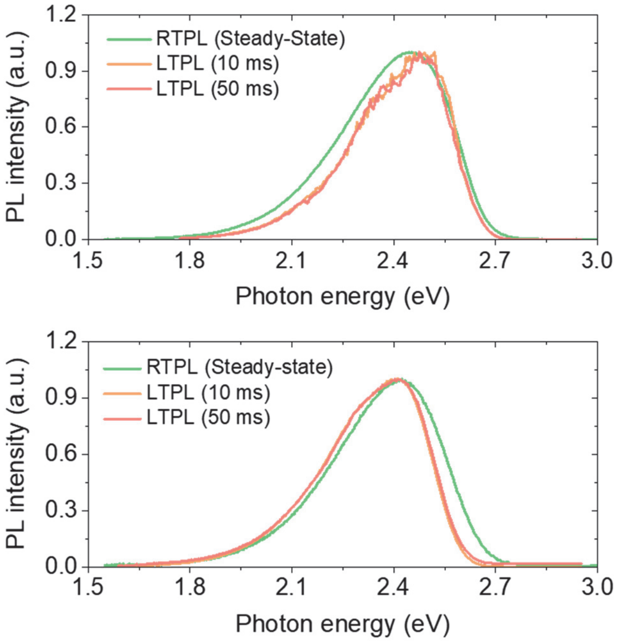

Supplementary Fig. 1 | The RTPL profiles (at steady-state) and LTPL profiles (at 77*K*) profiles for 4CzIPN are presented for the solution (Top, conc. 0.05 mM, toluene) and solid-state (Bottom, 5.0 wt.% doped *m*CBP host), respectively. The unit is arbitrary (arbitrary unit, a.u.).

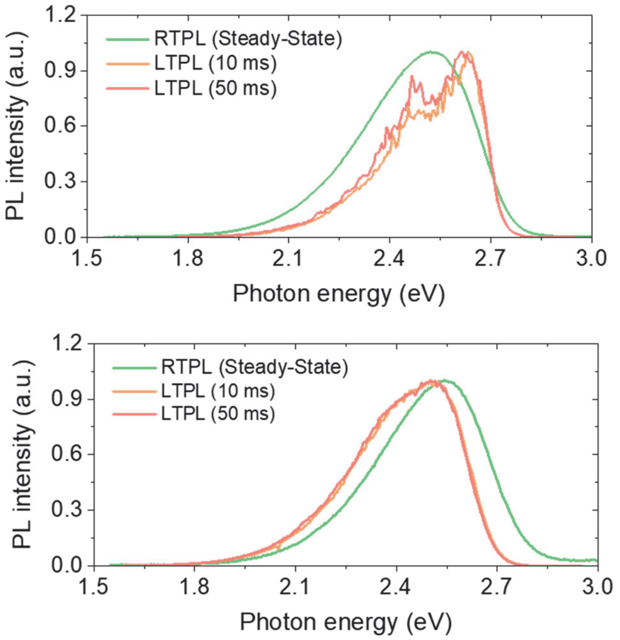

Supplementary Fig. 2 | The RTPL profiles (at steady-state) and LTPL profiles (at 77*K*) profiles for *o*-3CzIPN are presented for the solution (Top, conc. 0.05 mM, toluene) and solid-state (Bottom, 5.0 wt.% doped *m*CBP host), respectively. The unit is arbitrary (arbitrary unit, a.u.).

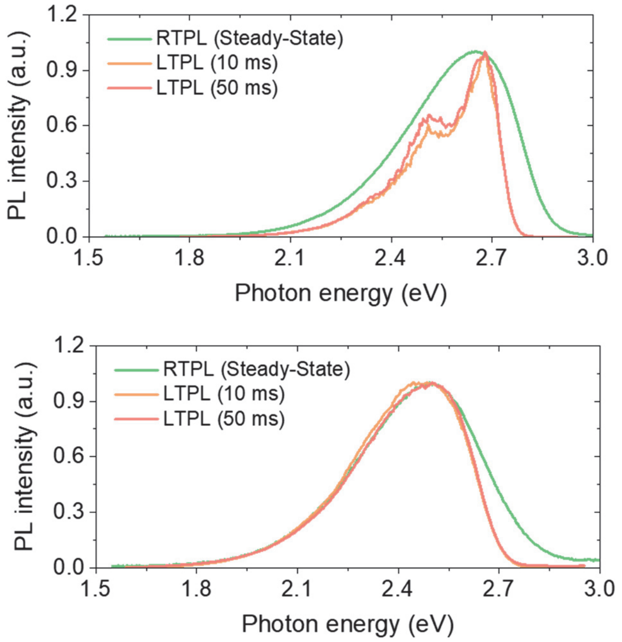

Supplementary Fig. 3 | The RTPL profiles (at steady-state) and LTPL profiles (at 77*K*) profiles for *m*-3CzIPN are presented for the solution (Top, conc. 0.05 mM, toluene) and solid-state (Bottom, 5.0 wt.% doped *m*CBP host), respectively. The unit is arbitrary (arbitrary unit, a.u.).

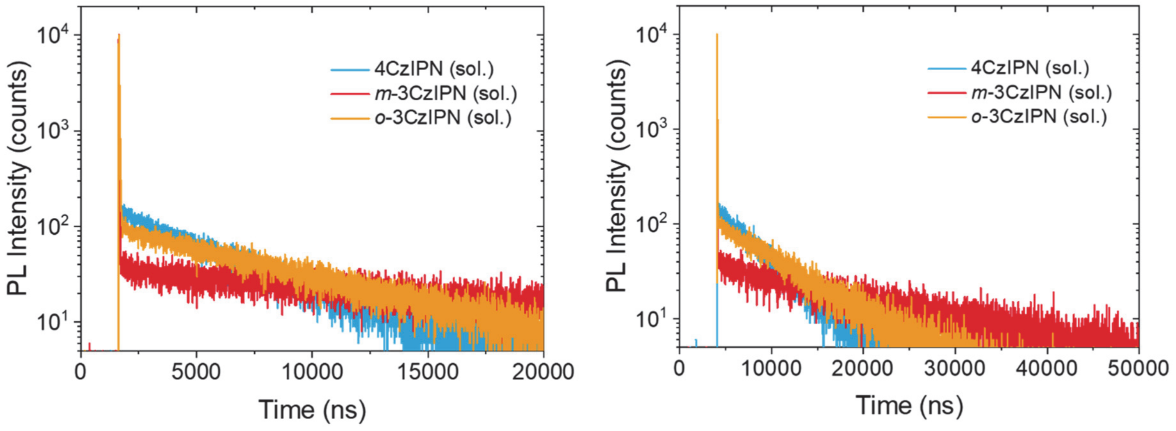

Supplementary Fig. 4 | The Tr-PL profiles for Ar-purged IPN derivatives diluted in toluene (conc. 0.05 mM) recorded at various time-scale [20 μs (left) and 50 μs (right), respectively].

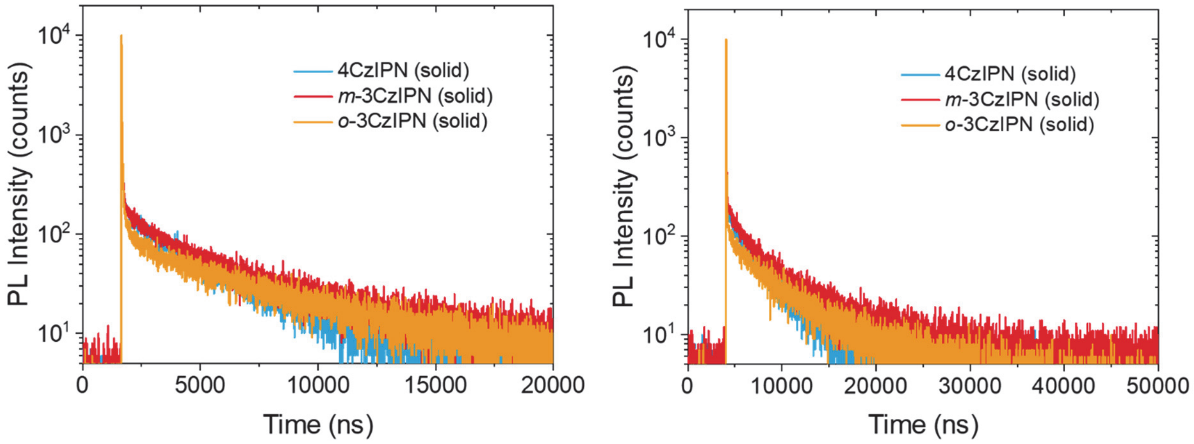

Supplementary Fig. 5 | The Tr-PL profiles for Ar-purged IPN derivatives doped on *m*CBP host film (conc. 5.0 wt.%, thickness: 50 nm) recorded at various time-scale [20 μs (left) and 50 μs (right), respectively].

D. Computational Results

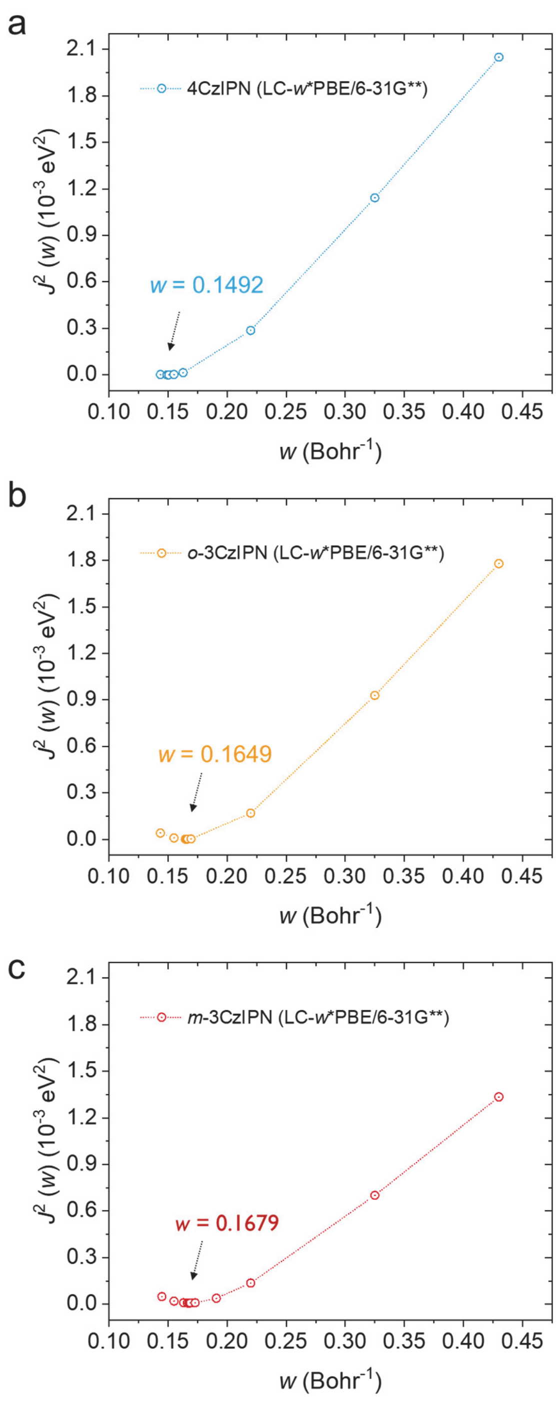

Supplementary Fig. 6 | Optimal *w** found using LC-w*PBE/6-31G** referencing from the optimized ground state geometry at the DFT level (B3LYP/6-31G**) for (a) 4CzIPN, (b) *o*-3CzIPN, and (c) *m*-3CzIPN, respectively.

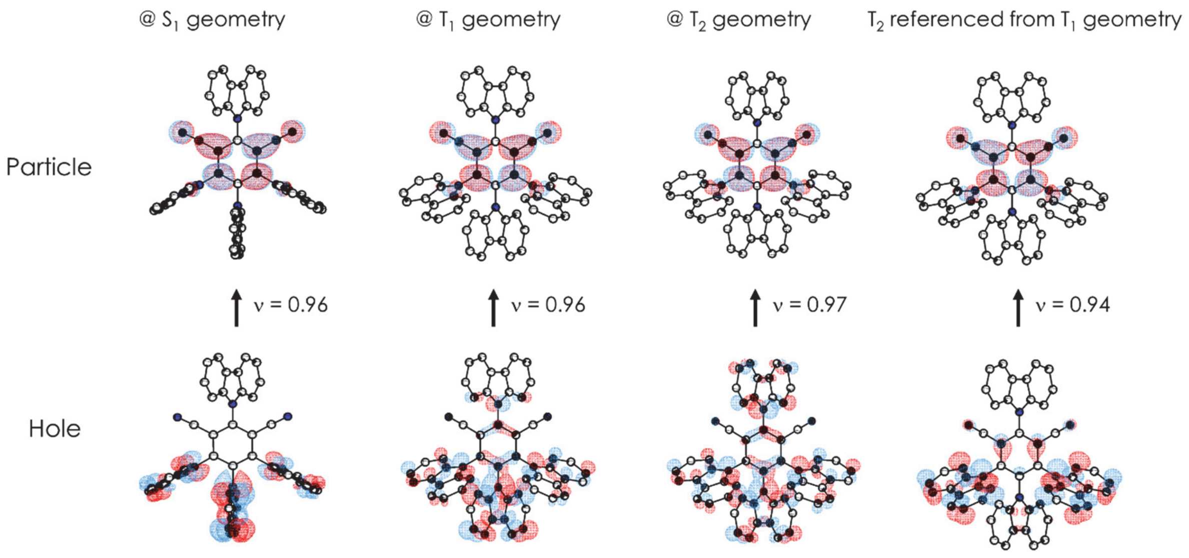

Supplementary Fig. 7 | The NTO pairs for the optimized S_1, T_1, and T_2 state (i.e., adiabatic) at the respective 4CzIPN geometry were obtained using TD-DFT within TDA (LC-w*PBE/6-31G**). Hole and particle wave functions with the weight (*v*) is placed.

Our results reveal that the equilibrated S_1 state of 4CzIPN exhibited the formation of ^{1}CT, which is due to the hole and particle densities being localized on the 1,3,4-position Cz units and IPN unit, respectively (**Supplementary Fig. 7** and **Fig. 3**a). Interestingly, the molecular orbital (MO) excitation configuration at the S_1 state looks like that of *o*-3CzIPN without the 2-Cz unit (as shown in **Supplementary Fig. 8**). This is because, the 2-Cz unit maintained a dihedral angle between the IPN unit even at the excitation from S_0 to S_1 state (i.e., 69.3° → 63.8°), while the other 1,3,4-position Cz units became orthogonal in both 4CzIPN and *o*-3CzIPN cases, leading to an out-of-plane conformation between 2-Cz and 4-Cz moiety in 4CzIPN (∠CNNC ≈ 28.5°).

Notably, in the T_1 and T_2 equilibrated geometries for the *o*-3CzIPN and *m*-3CzIPN molecules, they exhibited a common MO excitation shape, but their energy ordering of the MO character was completely inverted (refer to **Fig. 3**c). In particular, the hole density is strongly localized on the 1,3-position Cz and IPN units. All hole densities reside on 4,6-di(9*H*-carbazol-9-yl)isophthalonitrile (*m*-2CzIPN) molecular backbone in the T_1 and T_2 of *o*-3CzIPN, respectively, while it is opposite for those in *m*-3CzIPN (**Supplementary Fig. 9**). Here, we can find this specific MO triplet excitation is employed at the vertical T_2 transition referenced from the T_1 geometry of 4CzIPN as shown in **Supplementary Fig. 7**. Therefore, we can conclude that a specific triplet state with a common excitation MO shape represents the electronic structure of a partial molecular framework within the 4CzIPN molecule.

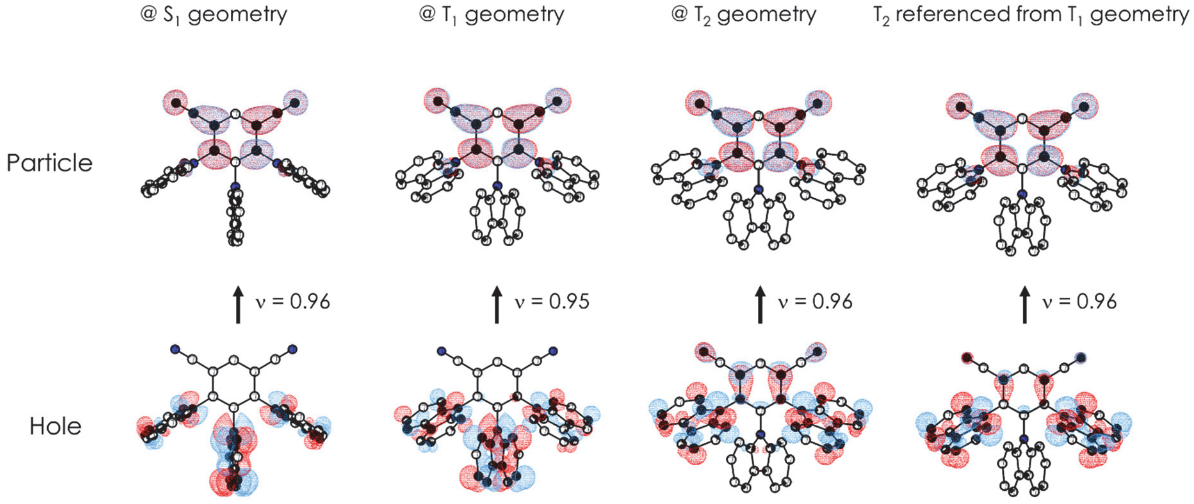

Supplementary Fig. 8 | The NTO pairs for the optimized S_1, T_1, and T_2 state (i.e., adiabatic) at the respective *o*-3CzIPN geometry were obtained using TD-DFT within TDA (LC-w*PBE/6-31G**). Hole and particle wave functions with the weight (*v*) is placed.

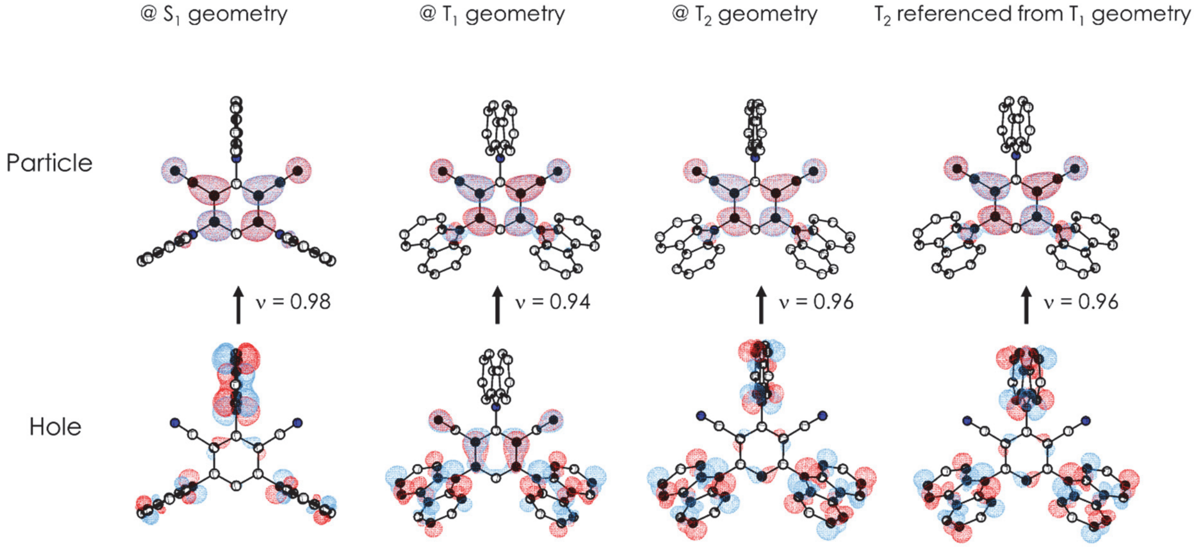

Supplementary Fig. 9 | The NTO pairs for the optimized S_1, T_1, and T_2 state (i.e., adiabatic) at the respective *m*-3CzIPN geometry were obtained using TD-DFT within TDA (LC-w*PBE/6-31G**). Hole and particle wave functions with the weight (*v*) is placed.

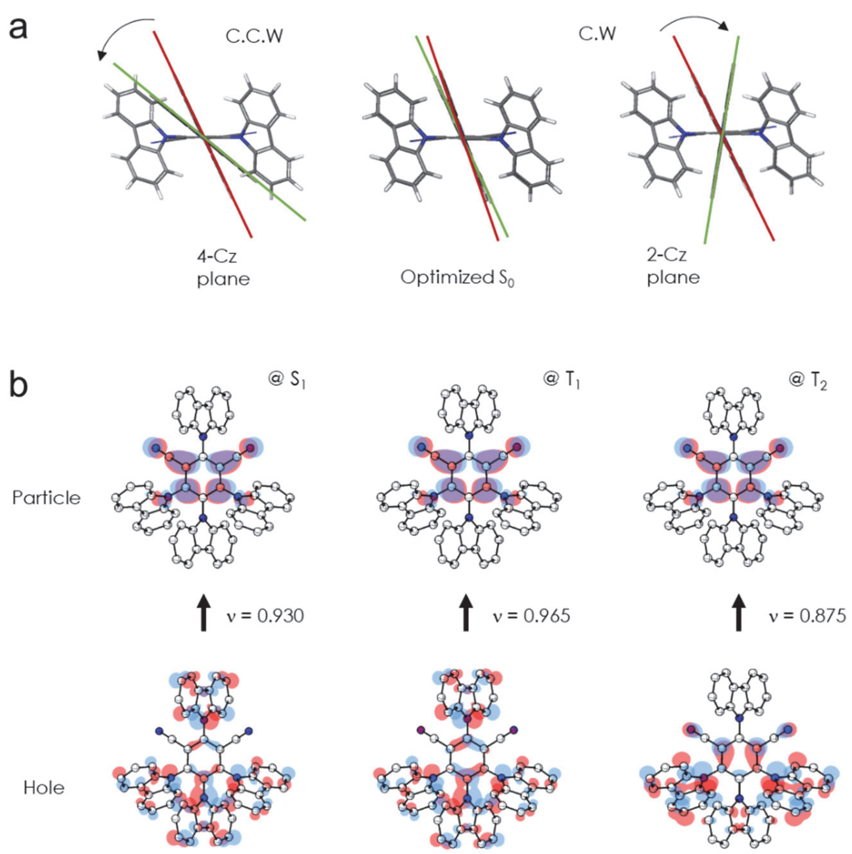

Supplementary Fig. 10 | (a) The dihedral angle changes between the 2-Cz and 4-Cz planes from the optimized ground state at DFT level (B3LYP/6-31G**). C.C.W stands for counterclockwise, and CW stands for clockwise. (b) The NTO pairs for the S_1, T_1, and T_2 states at the ground state geometry by using TD-DFT within TDA (LC-*w**PBE/6-31G**). Hole and particle wave functions with the weight (v) is placed.

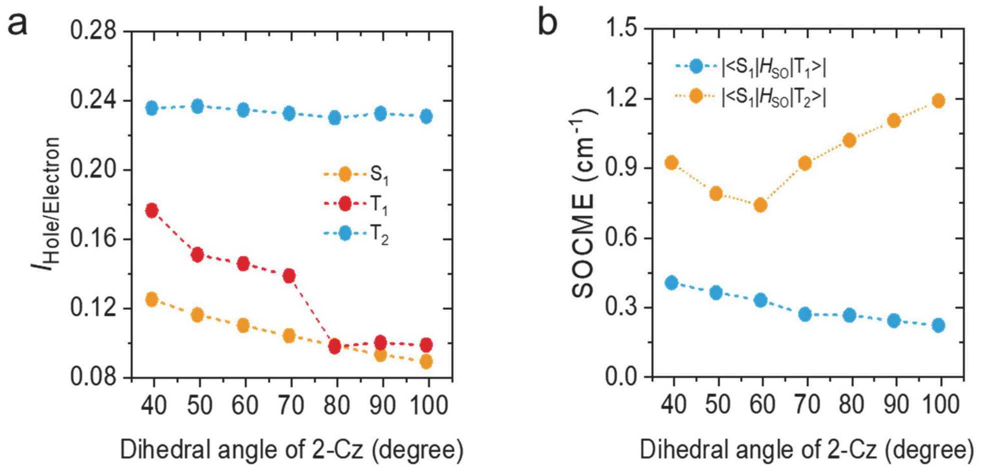

Supplementary Fig. 11 | (a) The extent of overlap ($I_{Hole/Electron}$) between hole and electron wave functions and (b) the spin-orbit coupling matrix element (SOCME) change of 4CzIPN molecule in accordance with the dihedral angle of 2-Cz (in steps of 10°). In this work, we defined the overlap function by using $I_{Hole/Electron} = \int min\,[\rho_{Hole}(r), \rho_{Electron}(r)]dr$.

To support our consideration, we carried out the quantum-mechanical/molecular-mechanical multi-scale (QM/MM) simulation to extend our theoretical modelling to condensed phase films, specifically a 5.0 wt.% doped *m*CBP film. (see 'Computational details' in SI and **Supplementary Figs. 11–18** for more information). In this simulation, we non-empirically determined the *w** values for each individual molecule (**Supplementary Fig. 12**), 4CzIPN, *o*-3CzIPN, and *m*-3CzIPN, at the snapshot from the converged frame and described the electronic structure distribution (**Supplementary Figs. 13–15**), obtained from the respective single-point results for each molecular geometry.

Strikingly, our findings reveal that, if the electronic structure distributions for T_1 and T_2 states obtained from molecules are statistically treated as a system (assigned to $T_{Cluster}$), it can effectively mimic the LTPL responses collected in the film state (refer to **Supplementary Figs. 1–3**). Consequently, we can provide the theoretical evidence that the presence of two distinct electronic transitions in solution-state (toluene, conc. 0.05 mM), particularly in the case of *o*-3CzIPN and *m*-3CzIPN, may depend on the similarity of MO excitation characters between the T_1 and T_2 states, giving rise to the efficient internal conversion (IC) rate (k_{IC}).

In other words, 4CzIPN has a considerable coupling between ^{3}CT at T_1 and ^{3}LE at T_2 state even at toluene (**Supplementary Fig. 7**), which differentiates from those of its partial molecules with the decoupled electronic transition. We further examined the rotational freedom of the 1,2,3,4 positions in the condensed solid phase, as shown in **Supplementary Figs. 16–17**. Notably, all models consistently indicate that an available torsional motion of the 2-Cz unit could act as a driving force for an efficient spin-flip process even in the solid phase.

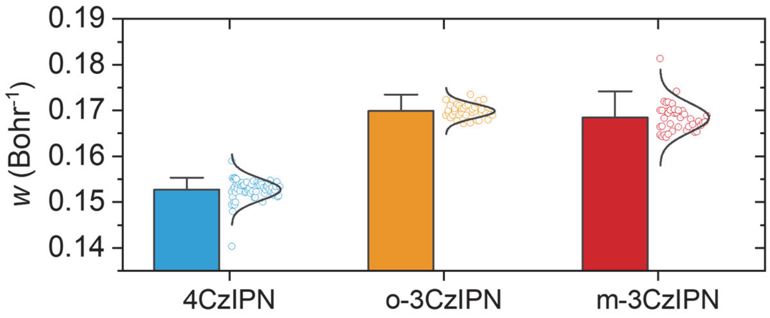

Supplementary Fig. 12 | The tuned *w** distribution of respective geometry of IPN-derivative cluster extracted from the converged snapshot (@ 100 ns, equilibrated state).

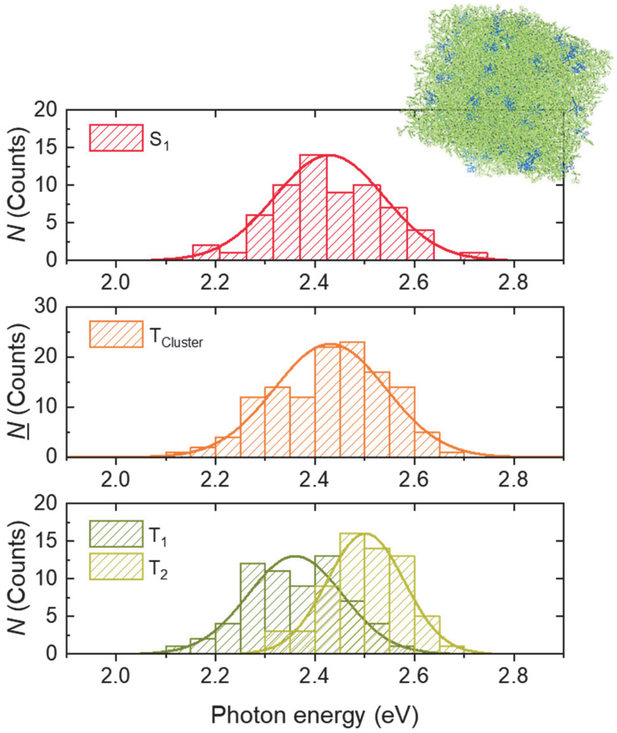

Supplementary Fig. 13 | The S_1, T_1, T_2, and $T_{Cluster}$ distributions for the solid-state model, 5.0 wt.% doped 4CzIPN on *m*CBP host (inset: the equilibrated solid-state by MD simulation @ 100.0 ns). In this work, we tuned the value of *w** for the respective geometry of the 4CzIPN cluster extracted from the snapshot (@ 100.0 ns, equilibrated state). With this optimal *w**, the electron structures of 4CzIPN clusters at the equilibrated state were investigated by using TD-DFT within TDA (LC-*w**PBE/6-31G**).

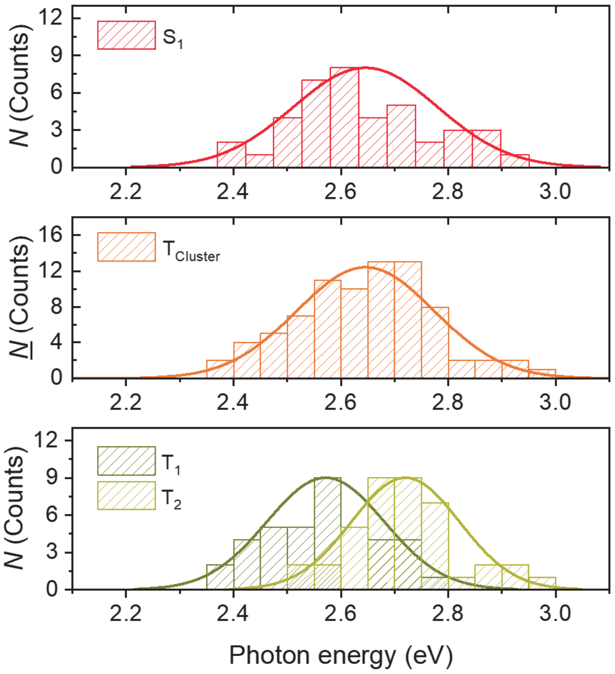

Supplementary Fig. 14 | The S_1, T_1, T_2, and $T_{Cluster}$ distributions for the solid-state model, 5.0 wt.% doped *o*-3CzIPN on *m*CBP host. In this work, we tuned the value of *w** for the respective geometry of the *o*-3CzIPN cluster extracted from the snapshot (@ 100.0 ns, equilibrated state). With this optimal *w** (refer to **Supplementary Fig. 12**), the electron structures of *o*-3CzIPN clusters at the equilibrated state were investigated by using TD-DFT within TDA (LC-*w**PBE/6-31G**).

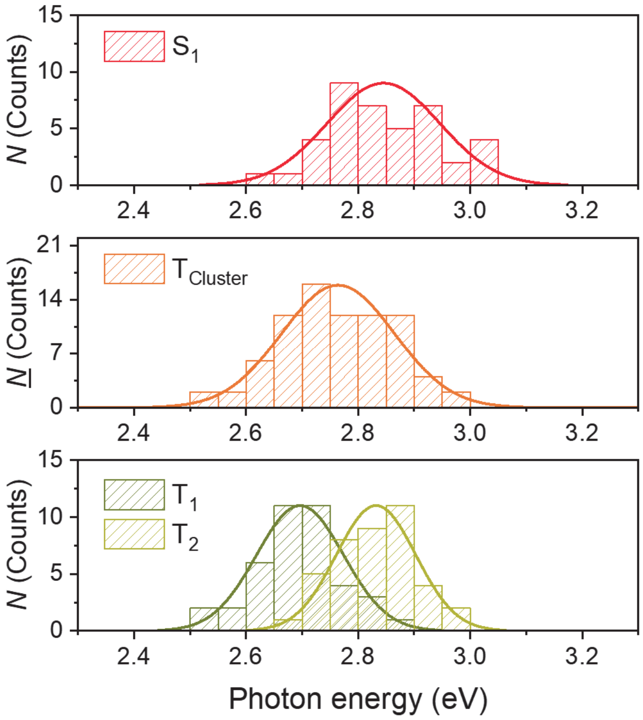

Supplementary Fig. 15 | The S_1, T_1, T_2, and $T_{Cluster}$ distributions for the solid-state model, 5.0 wt.% doped *m*-3CzIPN on *m*CBP host. In this work, we tuned the value of *w** for the respective geometry of the *m*-3CzIPN cluster extracted from the snapshot (@ 100.0 ns, equilibrated state). With this optimal *w** (refer to **Supplementary Fig. 12**), the electron structures of *m*-3CzIPN clusters at the equilibrated state were investigated using TD-DFT within TDA (LC-*w**PBE/6-31G**).

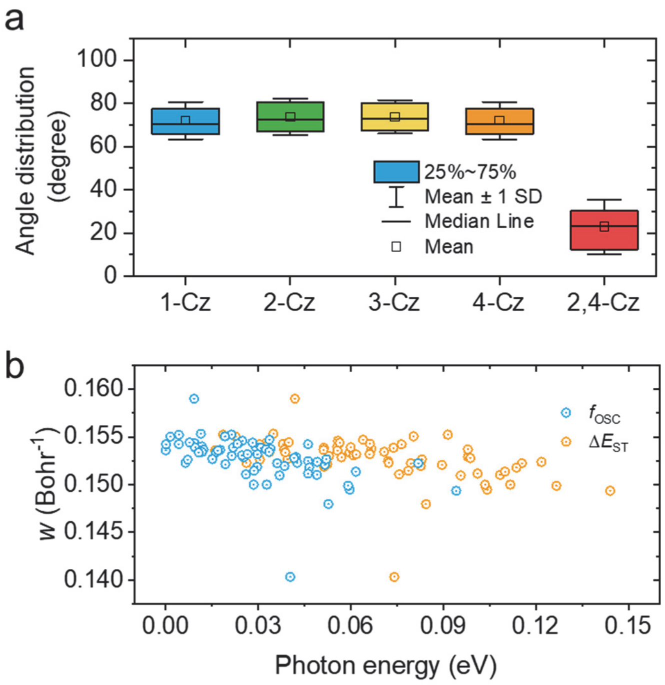

Supplementary Fig. 16 | (a) The dihedral angle distributions of 4CzIPN molecular clusters at converged-state (@ 100.0 ns). The error bar indicates 1.0 standard deviation (SD) from the median. We defined a series of angles between (btw) two planes [i.e., *N*-Cz btw IPN] formed by three atoms for the first plane (i.e., IPN moiety in this study) and those for the second plane (i.e., *N*-Cz where *N* is 1, 2, 3, and 4, respectively). (b) The oscillator strength (f_{OSC}) and ΔE_{ST} distributions for respective 4CzIPN geometries calculated with tuned w^*.

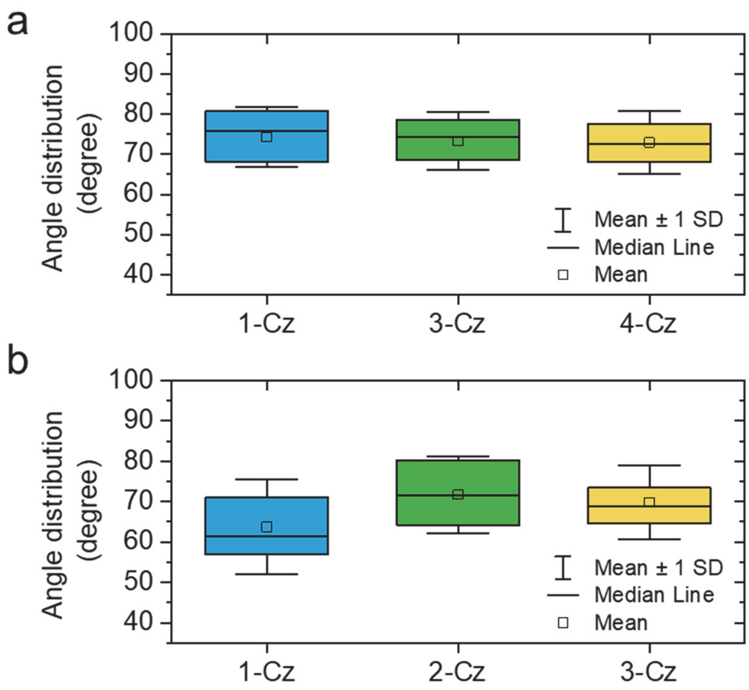

Supplementary Fig. 17 | The dihedral angle distributions of (a) *o*-3CzIPN and (b) *m*-3CzIPN clusters at the converged state (the snapshot @ 100.0 ns) obtained from MD simulations.

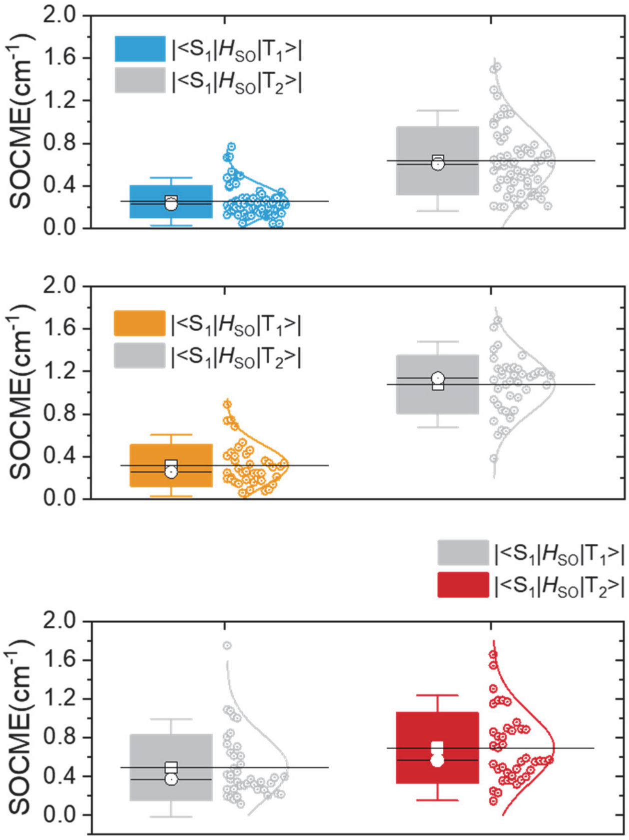

Supplementary Fig. 18 | The SOCME distribution at each condensed film state model for 4CzIPN (Top), *o*-3CzIPN (Middle), and *m*-3CzIPN (Bottom) doped system, respectively. The error bar indicates 1.0 standard deviation (SD) from the median (square). The circle symbol represents the mean in all cases.

E. Rate Relation Based on Exciton Dynamics

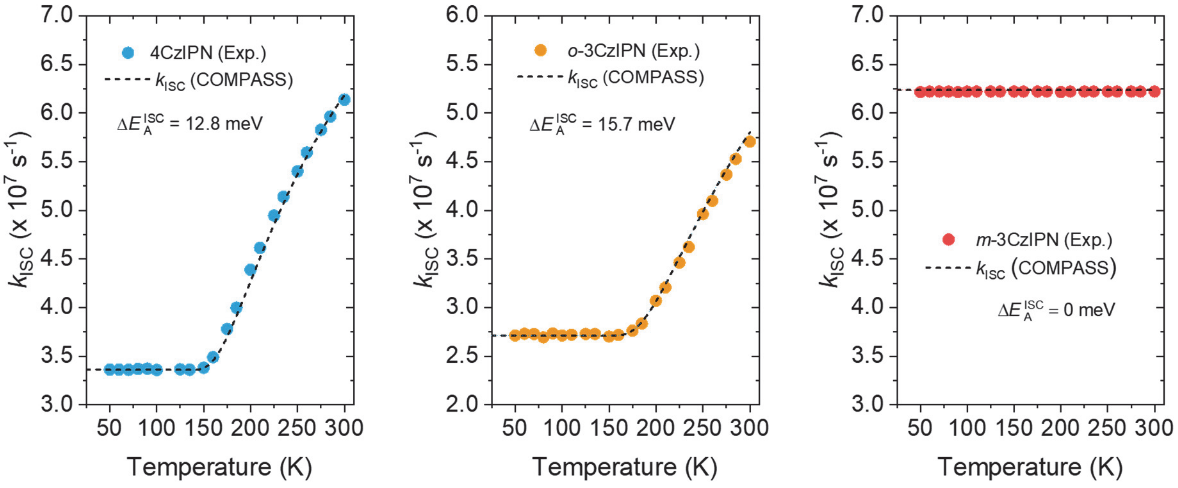

Supplementary Fig. 19 | Temperature dependence of the total (effective) intersystem crossing rate constant ($k_{\mathrm{ISC}}^{\mathrm{eff}}$) from S_1 state to T_1 and T_2 states for IPN derivatives (i.e., 4CzIPN, *o*-3CzIPN, and *m*-3CzIPN) using **Eqs.** (S15) and (6).

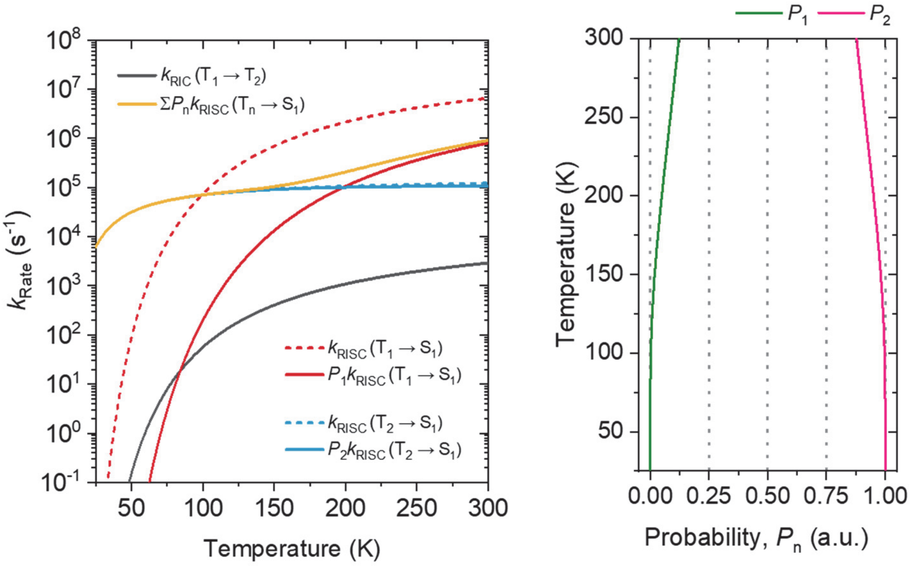

Supplementary Fig. 20 | The calculated rate constants (i.e., $k_{\mathrm{RIC}}^{\mathrm{T_1 \rightarrow T_2}}$, $k_{\mathrm{RISC}}^{\mathrm{T_1 \rightarrow S_1}}$, $k_{\mathrm{RISC}}^{\mathrm{T_2 \rightarrow S_1}}$, and k_{RISC}^{eff}) of *m*-3CzIPN doped system (left) and the probability (P_n) of the system occupying the electronic-state *n* in accordance with the temperature (right)

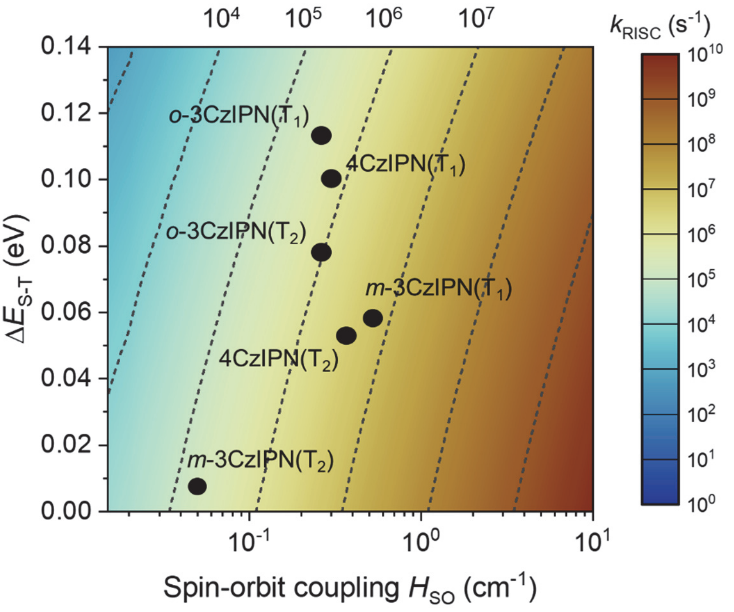

Supplementary Fig. 21 | Theoretical kinetic prediction of k_{RISC} as a function of H_{SO} and $\Delta E_{\mathrm{S\text{-}T}}$ with fixed reorganization energy (0.2 eV) and temperature (300 *K*) under the framework of Fermi's golden rule. Hereby, we used $k_{\mathrm{RISC}}^{\mathrm{T}_n\to\mathrm{S}_1} = \frac{2\pi}{\hbar}\left|\langle T_{\mathrm{n}}|\hat{H}_{\mathrm{SO}}|S_1\rangle\right|^2 \rho_{\mathrm{FC}}(T)$, where $\langle T_{\mathrm{n}}|\hat{H}_{SO}|S_1\rangle$ denotes the SOCME between the S_1 and the excited triplet (T) states, including the high-lying triplet state (i.e., T_2 state, *n* = 2), and ρ_{FC} is the Franck–Condon-weighted density of states.

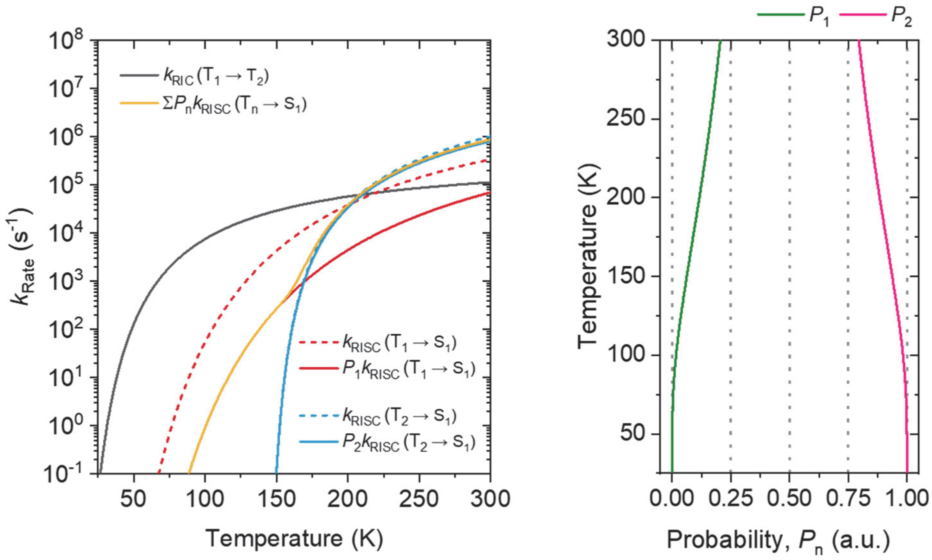

Supplementary Fig. 22 | The calculated rate constants (i.e., $k_{\mathrm{RIC}}^{\mathrm{T}_1\to\mathrm{T}_2}$, $k_{\mathrm{RISC}}^{\mathrm{T}_1\to\mathrm{S}_1}$, $k_{\mathrm{RISC}}^{\mathrm{T}_2\to\mathrm{S}_1}$, and k_{RISC}^{eff}) of *o*-3CzIPN doped system (left) and the probability (P_n) of the system occupying the electronic-state *n* in accordance with the temperature (right)

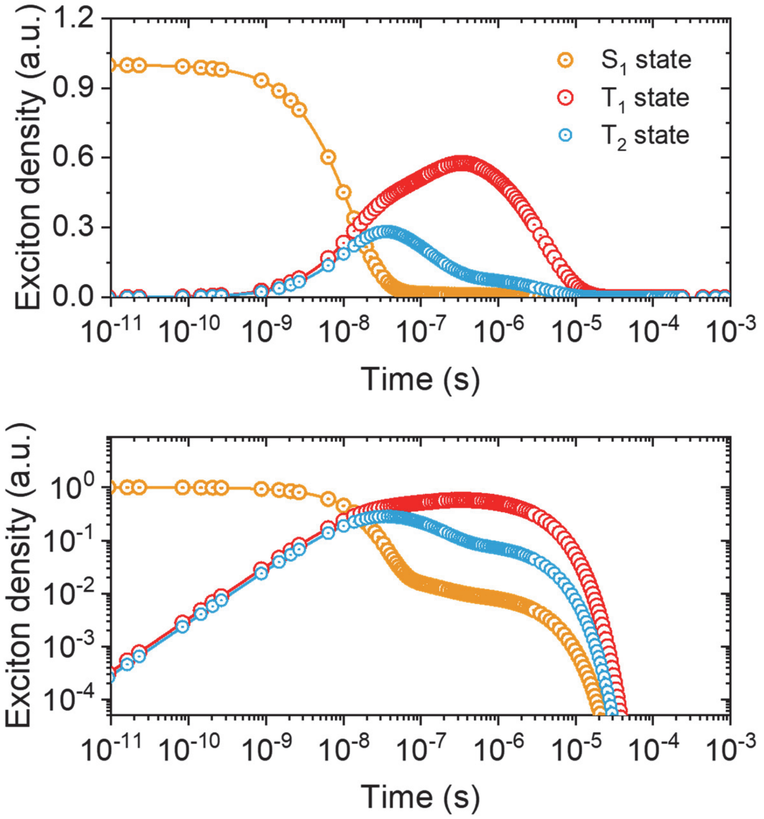

Supplementary Fig. 23 | Time-dependent relative exciton density changes at S_1, T_1, and T_2 states for 4CzIPN by after the optical excitation. The solid (symbol) denotes the analytical (numerical) solution of the optical COMPASS model at *RT*, respectively [refer to **Eqs.** (S9) to (S11)]. The unit is arbitrary (arbitrary unit, a.u.).

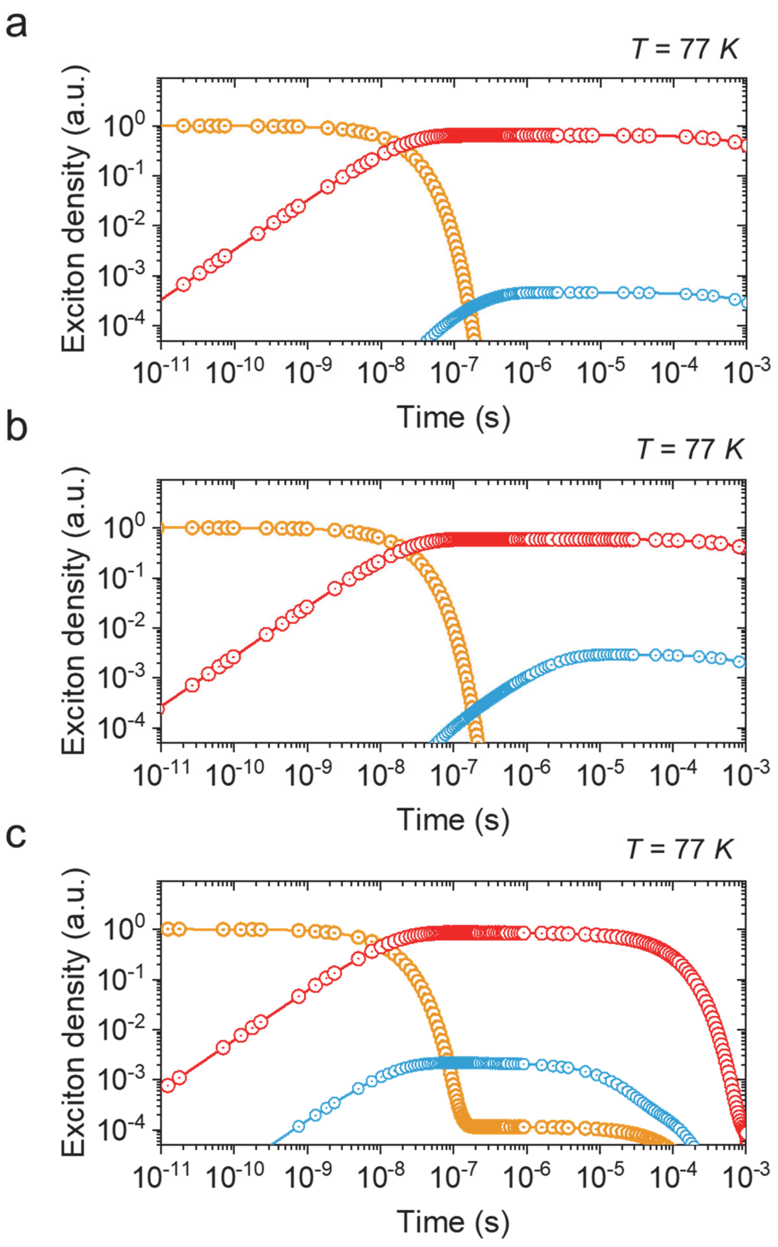

Supplementary Fig. 24 | Time-dependent relative exciton density changes at S_1, T_1, and T_2 states for (a) 4CzIPN, (b) *o*-3CzIPN, and (c) *m*-3CzIPN after the optical excitation. The solid (symbol) denotes the analytical (numerical) solution of the COMPASS model at 77*K*, respectively [refer to **Eqs.** (S9) to (S11)]. The unit is arbitrary (arbitrary unit, a.u.).

F. Device Characterization

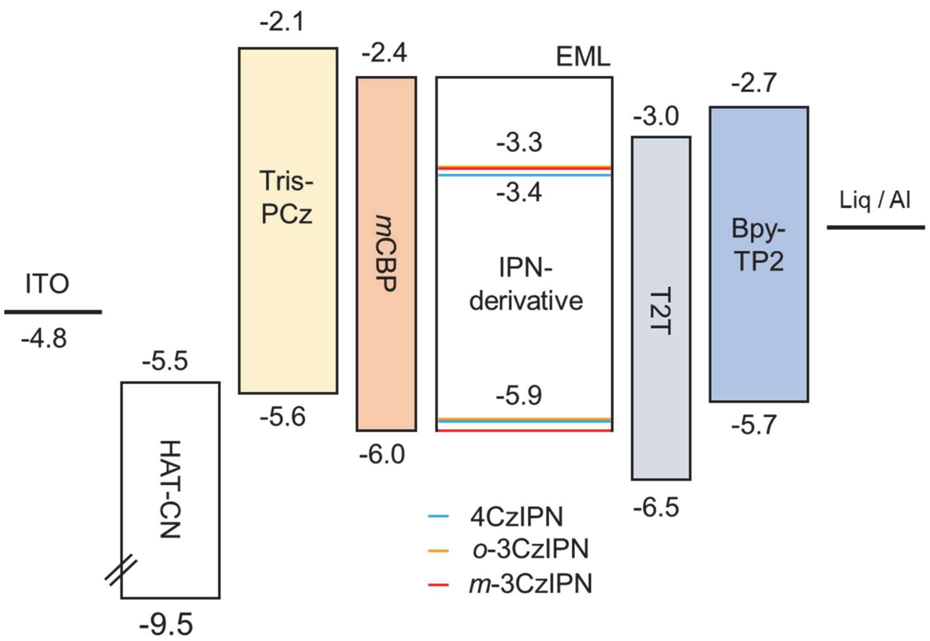

Supplementary Fig. 25 | The proposed OLED device architecture for the IPN series in this study. Herein, we determined the value of HOMO and LUMO levels for IPN derivatives (4CzIPN, *o*-3CzIPN, and *m*-3CzIPN) by measuring the neat film sample deposited on the quartz substrate. The measured HOMO level using the photoelectron spectrometer (AC-3, Riken Keiki) for 4CzIPN, *o*-3CzIPN, *m*-3CzIPN was 5.90, 5.87, and 6.02 eV, respectively.

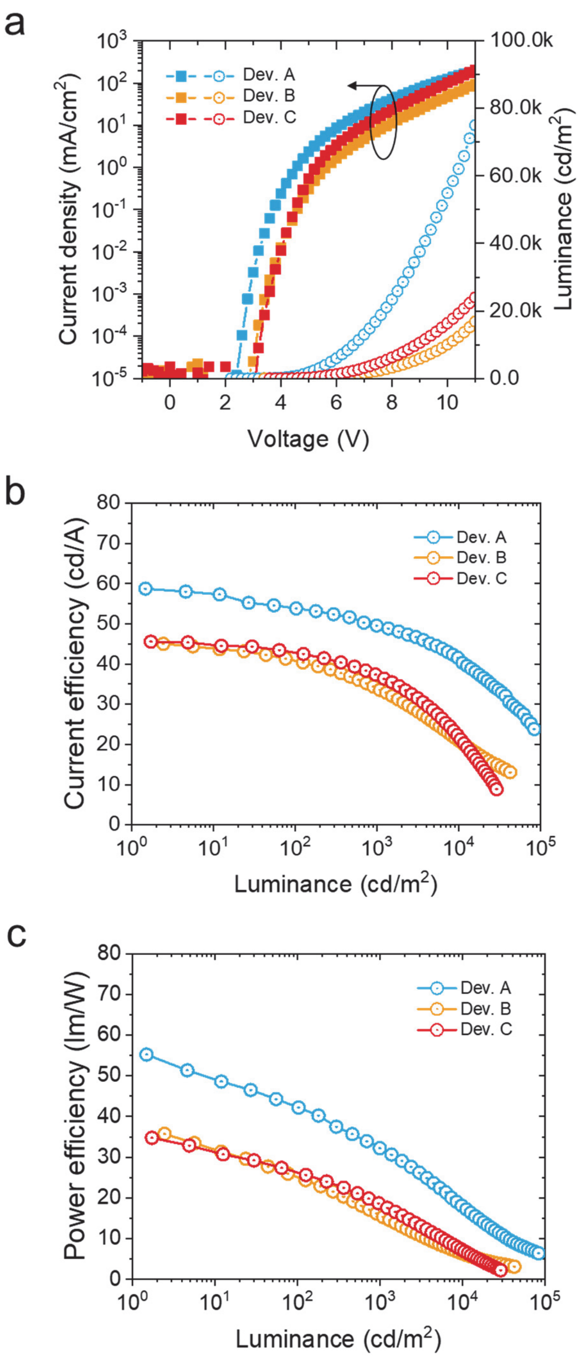

Supplementary Fig. 26 | (a) *J*-*V*-*L* characteristics (b) the current efficiency- and (c) power efficiency curves *vs.* luminance for Dev. A (4CzIPN), Dev. B (*o*-3CzIPN) and Dev. C (*m*-3CzIPN), respectively.

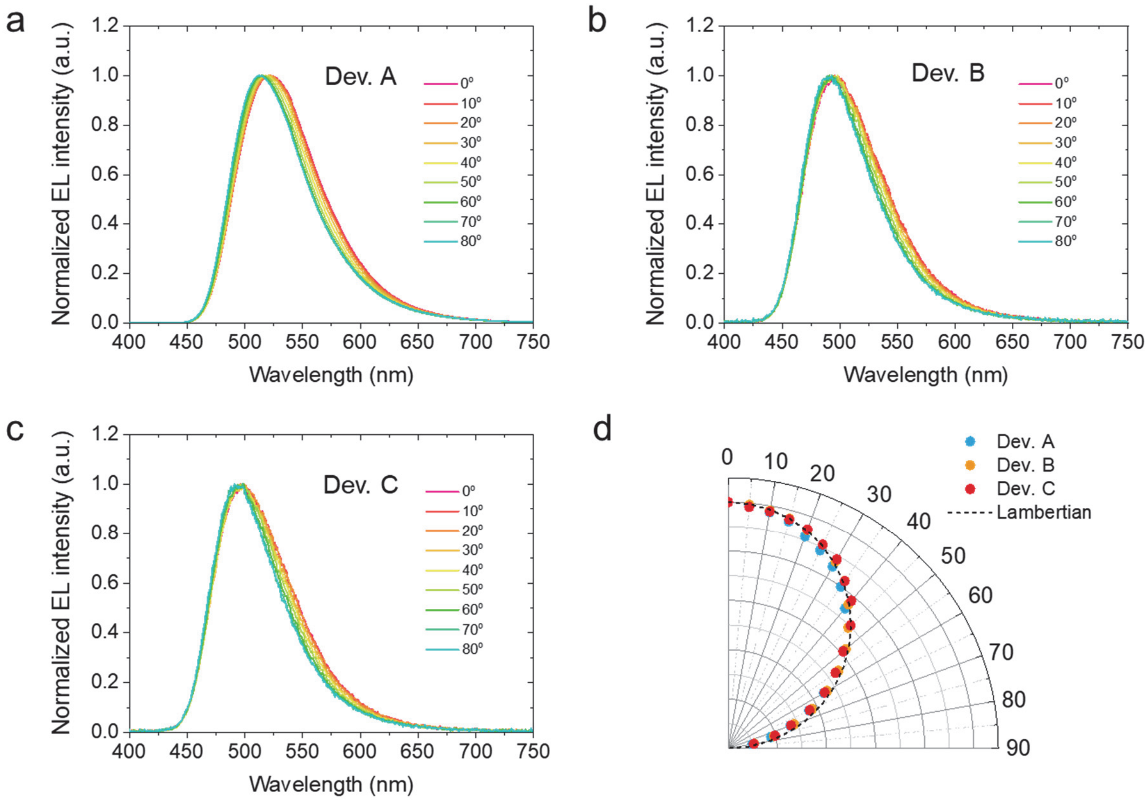

Supplementary Fig. 27 | Angular EL spectra for (a) Dev. A, (b) Dev. B, and (c) Dev. C, respectively. Here, we measured the angular EL spectra in steps of 5° but displayed them in 10-degree increments for figure clarity. (d) The normalized angular EL intensities for IPN derivatives.

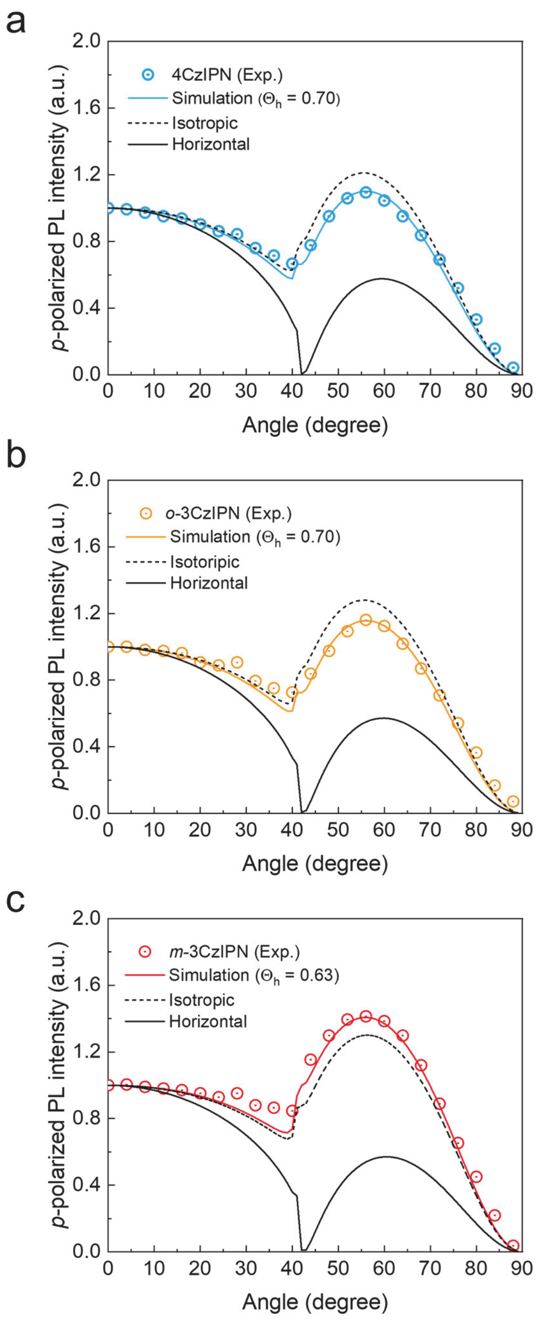

Supplementary Fig. 28 | Angular-dependent *p*-polarized PL profiles for (a) 4CzIPN (5.0 wt.% doped) (b) *o*-3CzIPN (5.0 wt.% doped), and (c) *m*-3CzIPN (5.0 wt.% doped) in *m*CBP host, respectively. Here, we obtained the angle-dependent *p*-polarized PL profiles in steps of 2° but displayed them in 4-degree increments for the clarity of figures.

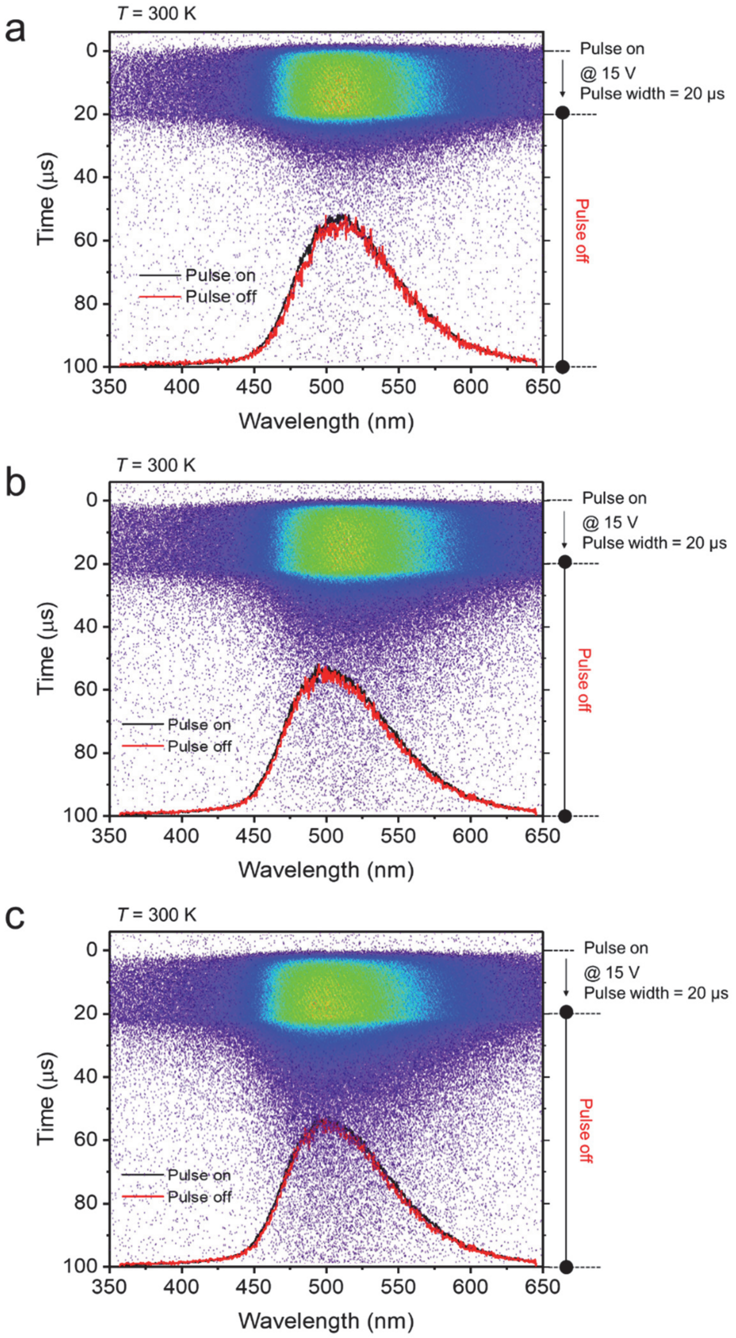

Supplementary Fig. 29 | The recorded Tr-EL decay response for (a) Dev. A (4CzIPN) (b) Dev. B (*o*-3CzIPN), and (c) Dev. C (*m*-3CzIPN) in this study.

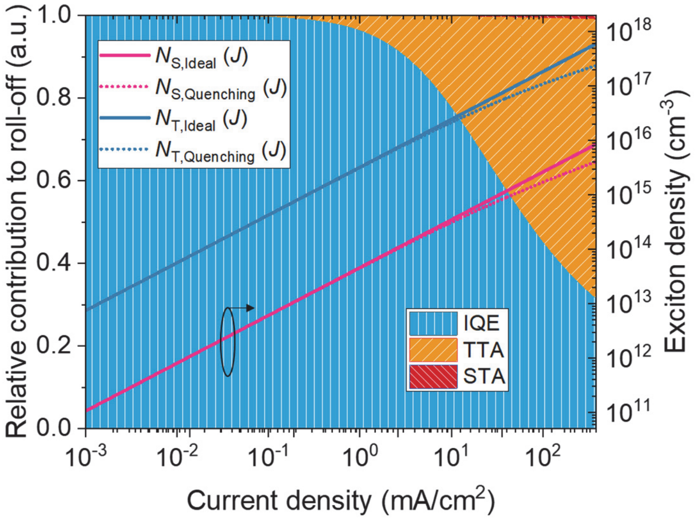

Supplementary Fig. 30 | Simulated relative contributions of TTA- (orange) and STA-based quenching (red) to efficiency roll-off as a function of current density for Dev. B (*o*-3CzIPN).

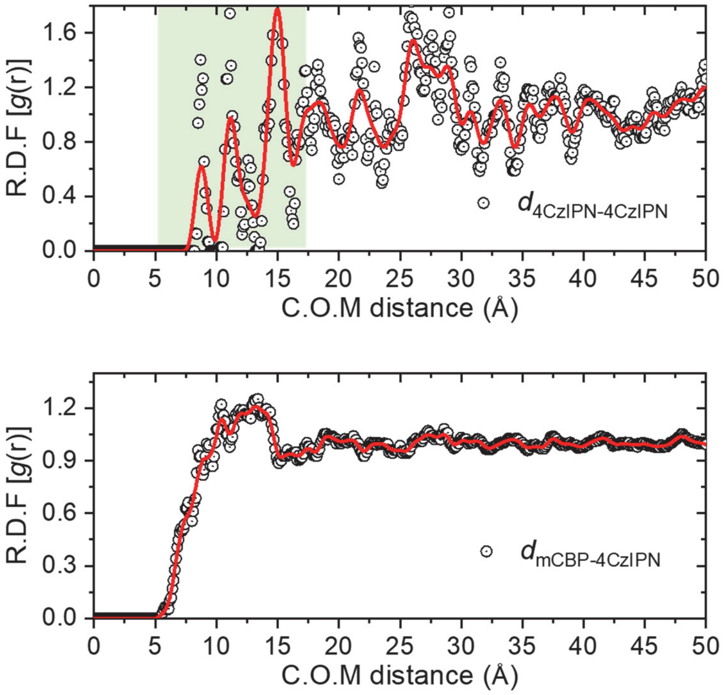

Supplementary Fig. 31 | The radial distribution functions (RDFs) for a 5.0 wt.% 4CzIPN doped *m*CBP binary film, as a function of the center of mass (C.O.M) intermolecular distances for 4CzIPN-4CzIPN (Top, $d_{\mathrm{4CzIPN-4CzIPN}}$) and *m*CBP-4CzIPN (Bottom, $d_{\mathrm{mCBP-4CzIPN}}$), respectively. Note that the system was analyzed by integrating the equilibrated-state in the time range of 80-100 ns.

G. Operational PL/EL Stability Test

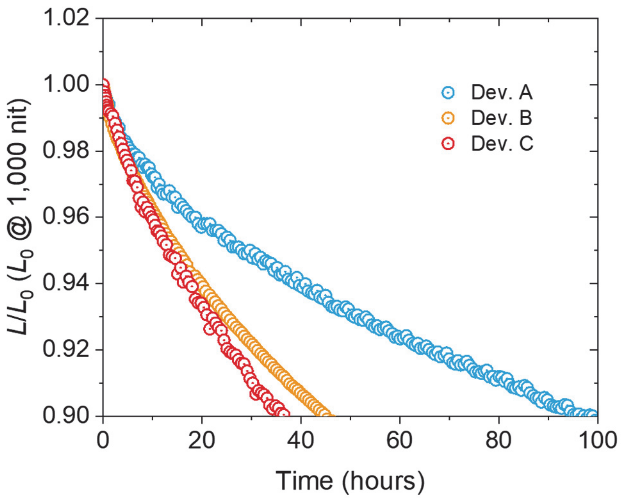

Supplementary Fig. 32 | The operational EL stability of the tested OLED devices at an initial luminance (L_0) of 1,000 nits.

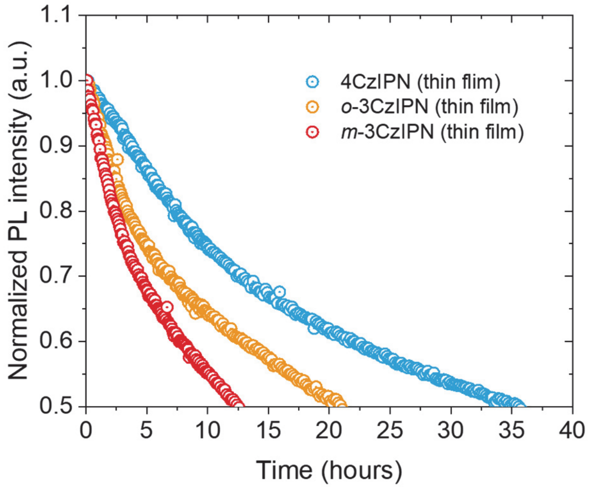

Supplementary Fig. 33 | The photostability of IPN-derivatives, with the same EML architecture (5.0 wt.% doped *m*CBP host), was investigated in this study. For the UV irradiation PL stability test, we used an excitation power of 5 mW/cm^2 at a wavelength of 340 ± 5 nm.

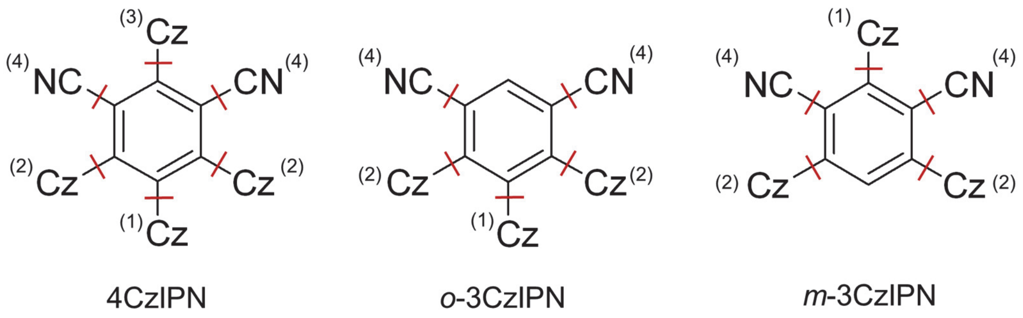

Supplementary Fig. 34 | The bond dissociation enthalpies (BDE) of 4CzIPN and its partial molecules, with a red solid line indicating the detached point, particularly for the rotational C-N bond. Here, we used LC-*w**PBE/6-31G** for the BDE calculation at the TD-DFT level within TDA.

Supplementary Table. 1 | The photophysical properties of IPN-derivative (solution)

Tr-PL scale	IPN-derivative	$^a\lambda_{peak}$ (nm)	k_{PF} (×10^7 s^{-1})	k_{DF} (×10^5 s^{-1})	τ_{PF} (×10^{-8} s)	τ_{DF} (×10^{-6} s)	Φ_{PLQY} (a.u.)	$^b\chi^2$
20 μs	4CzIPN	507	6.75	2.25	1.48	4.45	0.87	1.08
	o-3CzIPN	492	4.86	1.38	2.06	7.27	0.91	1.09
	m-3CzIPN	468	9.45	0.51	1.06	19.79	0.85	1.05
50 μs	4CzIPN	507	6.43	2.11	1.56	4.75	0.87	1.08
	o-3CzIPN	492	4.73	1.37	2.12	7.31	0.91	1.08
	m-3CzIPN	468	9.09	0.58	1.10	17.36	0.85	1.12

[a]The steady-state PL spectra at the solution-state (conc. 0.05 mM, toluene).
[b]Note that we hereby utilized the least squares fitting method for lifetime analysis. The analysis formula is as follows: $\chi^2 = \sum_{j=n_1}^{n_2} \frac{\left(I(t_j)-F(t_j)\right)^2}{I(t_j)} / (n_2 - n_1 + 1)$ where n_1 and n_2 are first and the last channels in the range selected for analysis. $I(t_j)$ is the count on each channel and $F(t_j)$ represents the corresponding fitted value.

Supplementary Table. 2 | The rate constants of IPN-derivative based on the three-level model (solution)

Tr-PL scale	IPN-derivative	k_r^S (×10^7 s^{-1})	k_{ISC} (×10^7 s^{-1})	k_{RISC} (×10^6 s^{-1})	k_{nr}^T (×10^4 s^{-1})	Φ_{DF}/Φ_{PF} (a.u.)	Φ_{ISC} (a.u.)	Φ_{RISC} (a.u.)
20 μs	4CzIPN	1.40	5.36	0.91	3.74	3.20	0.79	0.96
	o-3CzIPN	1.24	3.62	0.48	1.62	2.58	0.75	0.97
	m-3CzIPN	1.90	7.55	0.21	0.93	3.24	0.80	0.96
50 μs	4CzIPN	1.43	4.99	0.78	3.58	2.89	0.78	0.96
	o-3CzIPN	1.22	3.51	0.47	1.62	2.54	0.74	0.97
	m-3CzIPN	1.71	7.38	0.25	1.04	3.54	0.81	0.96

Supplementary Table. 3 | The photophysical properties of IPN-derivatives (solid-state)

Tr-PL scale	IPN-derivative	$^a\lambda_{peak}$ (nm)	k_{PF} [x10^7 s^{-1}]	k_{DF} [×10^5 s^{-1}]	τ_{PF} [×10^{-8} s]	τ_{DF} [×10^{-6} s]	Φ_{PLQY} [a.u.]	$^b\chi^2$
20 μs	4CzIPN	512	7.98	3.04	1.25	3.29	0.87	1.11
	o-3CzIPN	488	6.72	2.14	1.49	4.68	0.97	1.13
	m-3CzIPN	497	7.33	2.00	1.36	4.99	0.73	1.27
50 μs	4CzIPN	512	7.09	2.74	1.41	3.65	0.87	1.10
	o-3CzIPN	488	6.51	1.89	1.54	5.29	0.97	1.21
	m-3CzIPN	497	6.82	1.43	1.47	6.99	0.73	1.21

[a]The steady-state PL spectra measured at the solid-state (5.0 wt.% doped on *m*CBP host).
[b]Note that we hereby utilized the least squares fitting method for lifetime analysis. The analysis formula is as follows: $\chi^2 = \sum_{j=n_1}^{n_2} \frac{\left(I(t_j)-F(t_j)\right)^2}{I(t_j)} / (n_2 - n_1 + 1)$ where n_1 and n_2 are first and the last channels in the range selected for analysis. $I(t_j)$ is the count on each channel and $F(t_j)$ represents the corresponding fitted value.

Supplementary Table. 4 | The rate constants of IPN-derivative based on the three-level model (solid-state)

Tr-PL scale	IPN-derivative	k_r^S ($\times 10^7$ s^{-1})	k_{ISC} ($\times 10^7$ s^{-1})	k_{RISC} ($\times 10^6$ s^{-1})	k_{nr}^T ($\times 10^4$ s^{-1})	Φ_{DF}/Φ_{PF} (a.u.)	Φ_{ISC} (a.u.)	Φ_{RISC} (a.u.)
20 μs	4CzIPN	1.83	6.15	1.09	5.28	2.78	0.77	0.95
	o-3CzIPN	1.89	4.83	0.73	0.89	2.46	0.72	0.99
	m-3CzIPN	1.12	6.22	0.90	6.38	3.80	0.85	0.93
50 μs	4CzIPN	1.64	5.45	0.98	4.78	2.74	0.77	0.95
	o-3CzIPN	1.86	4.65	0.64	0.79	2.40	0.72	0.99
	m-3CzIPN	1.02	5.80	0.65	4.54	3.88	0.85	0.94

Supplementary Table. 5 | Exciton decay rate, effective spin-flip rate constants, and energy differences for IPN sets (5.0 wt.% doped at *m*CBP host film) at 300 *K*

IPN-derivative	4CzIPN	*o*-3CzIPN	*m*-3CzIPN	Unit
k_1 (Theory)[a]	8.19	6.74	7.92	10^7 s^{-1}
k_2 (Theory)[a]	7.77	1.33	0.96	10^6 s^{-1}
k_3 (Theory)[a]	2.51	1.32	1.40	10^5 s^{-1}
k_1 (Approx)[b]	8.01	6.69	7.36	10^7 s^{-1}
k_2 (Approx)[c]	7.82	1.26	0.13	10^6 s^{-1}
$\Delta E_{\mathrm{A}}^{\mathrm{ISC}}$	12.80	15.70	~ 0	meV
$\Delta E_{\mathrm{S_1-T_1}}$	100	113	58	meV
$\Delta E_{\mathrm{S_1-T_2}}$	52	78	7	meV
$\Delta E_{\mathrm{T_2-T_1}}$	48	35	51	meV
k_{ISC}^{eff}	6.18	4.80	6.24	10^7 s^{-1}
k_{RISC}^{eff}	3.36	0.88	0.91	10^6 s^{-1}
$k_{\mathrm{r}}^{\mathrm{S_1 \rightarrow S_0}}$	1.83	1.89	1.12	10^7 s^{-1}
$k_{\mathrm{r}}^{\mathrm{T_n \rightarrow S_0}}$	5.28 (*n*=2)	0.89 (*n*=2)	0.97 (*n*=1)	10^4 s^{-1}

[a]Rate constant based on COMPASS model.
[b]The simplified rate constant based on $k_1 \cong k_{\mathrm{r}}^{\mathrm{S_1 \rightarrow S_0}} + k_{\mathrm{nr}}^{\mathrm{S_1 \rightarrow S_0}} + k_{\mathrm{ISC}}^{\mathrm{S_1 \rightarrow T_2}} + k_{\mathrm{ISC}}^{\mathrm{S_1 \rightarrow T_1}}$
[c]The simplified rate constant based on $k_2 \cong k_r^{\mathrm{T_2 \rightarrow S_0}} + k_{\mathrm{IC}}^{\mathrm{T_2 \rightarrow T_1}} + P_2 k_{\mathrm{RISC}}^{\mathrm{T_2 \rightarrow S_1}}$

Supplementary Table. 6 | The effective rate constants for IPN sets (5.0 wt.% doped *m*CBP film) based on the COMPASS model (at 300 *K*)

IPN-derivative	4CzIPN	*o*-3CzIPN	*m*-3CzIPN	Unit
$k_{\mathrm{r}}^{\mathrm{S_1 \to S_0}}$	1.83	1.89	1.12	10^7 s^{-1}
$k_{\mathrm{ISC}}^{\mathrm{S_1 \to T_1}}$	3.36	2.71	6.22	10^7 s^{-1}
$k_{\mathrm{ISC}}^{\mathrm{S_1 \to T_2}}$	2.82	2.09	0.02	10^7 s^{-1}
$k_{\mathrm{r}}^{\mathrm{T_1 \to S_0}}$	0.42	0.38	9.65	10^3 s^{-1}
$k_{\mathrm{RISC}}^{\mathrm{T_1 \to S_1}}$	0.70	0.34	6.60	10^6 s^{-1}
$k_{\mathrm{RIC}}^{\mathrm{T_1 \to T_2}}$	7.03	1.14	0.03	10^5 s^{-1}
$k_{\mathrm{r}}^{\mathrm{T_2 \to S_0}}$	5.28	0.89	~ 0	10^4 s^{-1}
$k_{\mathrm{IC}}^{\mathrm{T_2 \to T_1}}$	4.50	0.44	0.02	10^6 s^{-1}
$k_{\mathrm{RISC}}^{\mathrm{T_2 \to S_1}}$	3.78	1.02	0.12	10^6 s^{-1}

Supplementary Table. 7 | The calculated bond dissociation energies (eV) for a series of IPN derivatives at respective molecular geometry.

[a]State	Bond-type (Bond order)	Cliff point	4CzIPN	*o*-3CzIPN	*m*-3CzIPN
S_0 state	C-N (1)	(1)	4.49 eV	3.73 eV	3.83 eV
S_0 state	C-N (1)	(2)	4.56 eV	3.80 eV	3.89 eV
S_0 state	C-N (1)	(3)	4.64 eV	N/A	N/A
S_0 state	C-N (3)	(4)	5.75 eV	5.79 eV	5.74 eV
T_1 state	C-N (1)	(1)	2.19 eV	1.26 eV	1.23 eV
T_1 state	C-N (1)	(2)	2.26 eV	1.33 eV	1.29 eV
T_1 state	C-N (1)	(3)	2.34 eV	N/A	N/A
T_1 state	C-N (3)	(4)	3.45 eV	3.32 eV	3.14 eV
S_1 state	C-N (1)	(1)	2.15 eV	1.23 eV	1.21 eV
S_1 state	C-N (1)	(2)	2.21 eV	1.30 eV	1.27 eV
S_1 state	C-N (1)	(3)	2.30 eV	N/A	N/A
S_1 state	C-N (3)	(4)	3.40 eV	3.29 eV	3.12 eV
T_2 state	C-N (1)	(1)	2.07 eV	1.19 eV	1.14 eV
T_2 state	C-N (1)	(2)	2.13 eV	1.27 eV	1.21 eV
T_2 state	C-N (1)	(3)	2.22 eV	N/A	N/A
T_2 state	C-N (3)	(4)	3.32 eV	3.26 eV	3.06 eV

[a]The molecular geometries at ground state (S_0) are obtained from the LC-*w**PBE/6-31G** at DFT level. We then optimized the molecular geometries of S_1, T_1 and T_2 states by using tuned-LC-*w*PBE/6-31G** at the TD-DFT level within TDA.

References

1. Baleizao, C. & Berberan-Santos, M. N. Thermally activated delayed fluorescence as a cycling process between excited singlet and triplet states: application to the fullerenes. *J. Chem. Phys.* **126**, 204510 (2007).

2. Goushi, K., Yoshida, K., Sato, K. & Adachi, C. Organic light-emitting diodes employing efficient reverse intersystem crossing for triplet-to-singlet state conversion. *Nat. Photon*. **6**, 253-258 (2012).

3. Kobayashi T. et al. Contributions of a higher triplet excited state to the emission properties of a thermally activated delayed-fluorescence emitter. *Phys. Rev. Appl.* **7**, 034002 (2017).

4. Masui, K. Nakanotani, H. & Adachi, C. Analysis of exciton annihilation in high-efficiency sky-blue organic light-emitting diodes with thermally activated delayed fluorescence. *Org. Electron.* **14**, 2721-2726 (2013).

5. Kim, H. S., Lee, S. H. & Adachi, C. Comparison of Exciton Annihilation Processes in Prototypical Charge-Transfer Molecules. *J. Phys. Chem. C* ASAP (2023).
